# Targeting Adenosine A2b Receptor Promotes Penile Rehabilitation of Refractory Erectile Dysfunction

**DOI:** 10.1002/advs.202306514

**Published:** 2024-06-14

**Authors:** Yang Xiong, Feng Qin, Shanzun Wei, Xingliang Yang, Jun Li, Changjing Wu, Fuxun Zhang, Jiuhong Yuan

**Affiliations:** ^1^ Department of Urology and Andrology Laboratory West China Hospital Sichuan University Chengdu Sichuan 610041 China; ^2^ Division of Urology University of Texas Medical School at Houston Houston TX 77030 USA

**Keywords:** adenosine, A2b receptor, dipyridamole, penile rehabilitation, refractory erectile dysfunction

## Abstract

The mechanisms of adenosine and specific adenosine receptor subtypes in promoting penile rehabilitation remain unclear. Single‐cell RNA sequencing of human corpus cavernosum,  adenosine deaminase (ADA) and adenosine receptors knock‐out mice (ADA^−/−^, A1^−/−^, A2a^−/−^, A2b^−/−^, and A3^−/−^), and primary corpus cavernosum smooth muscle cells are used to determine receptor subtypes responsible for adenosine‐induced erection. Three rat models are established to characterize refractory erectile dysfunction (ED): age‐related ED, bilateral cavernous nerve crush related ED (BCNC), and diabetes mellitus‐induced ED. In single‐cell RNA sequencing data, the corpus cavernosum of ED patients show a decrease in adenosine A1, A2a and A2b receptors. In vivo, A2b receptor knock‐out abolishes adenosine‐induced erection but not that of A1, A2a, or A3 receptor. Under hypoxic conditions in vitro, activating the A2b receptor increases HIF‐1α and decreases PDE5 expression. In refractory ED models, activating the A2b receptor with Bay 60–6583 improves erectile function and down‐regulates HIF‐1α and TGF‐β. Administering Dipyridamole (40 mg Kg^−1^) to BCNC rats improve penile adenosine levels and erectile function. Our study reveals that the A2b receptor mediates adenosine‐induced penile erection. Activating the A2b receptor promotes penile rehabilitation of refractory ED by alleviating hypoxia and fibrosis.

## Graphical abstract

The A2b receptor of adenosine mediates adenosine‐induced penile erection, rather than the A1, A2a, and A3 receptors. Activating the A2b receptor promotes penile rehabilitation of refractory ED by alleviating hypoxia and fibrosis. Dipyridamole, an oral adenosine transporter inhibitor, can promote penile rehabilitation by attenuating hypoxia and fibrosis, showing therapeutic potential in clinics.

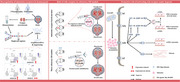

## 1 Introduction

Erectile dysfunction (ED) is a common clinical condition affecting 30–50% of males aged > 40 years.^[^ ^1^ ^]^ Heavy adverse impacts on patients’ mental health and quality of life demand effective treatment. At present, phosphodiesterase type 5 inhibitors (PDE5i) continue to be the preferred therapy for ED patients and have shown satisfactory effectiveness in the early stage. However, PDE5i still exerts no effect in some cases. The efficacy of PDE5i varies between 60–70% and even further decreases for refractory ED patients induced by diabetes or radical prostatectomy.^[^ ^2^, ^3^ ^]^ In addition, after one year, almost 50% of ED patients reported discontinuation of PDE5i.^[^ ^4^ ^]^ A lack of efficacy is one of the most important reasons.^[^ ^4^, ^5^ ^]^ Other therapies, such as intracavernosal injection of prostaglandin E and vacuum erectile devices are invasive and unpleasant. Thus, it is imperative to find new therapeutic targets and treatment strategies for ED.

Adenosine, like nitric oxide, is a vasodilator and has long been implicated in regulating penile erection.^[^ ^6^ ^]^ Previous studies have shown that adenosine can induce dose‐dependent increases in intracavernous pressure (ICP) and cause full erection of the penis.^[^ ^7^, ^8^ ^]^ Excess adenosine contributes to priapism.^[^ ^6^, ^8^ ^]^ Further experiments on mouse and human cavernous strips indicated that adenosine could induce penile relaxation by activating A2 receptors.^[^ ^9^, ^10^ ^]^ Moreover, it was found that an A2b receptor antagonist could abolish adenosine‐induce relaxation of cavernous strips from rabbits or priapism induced by sickle cell disease in mice.^[^ ^11^, ^12^ ^]^ These data suggest that the A2b receptor may be responsible for the pro‐erectile effect of adenosine. Conversely, Noto T et al. reported that a selective A2a receptor inhibitor could abolish adenosine‐induced erection.^[^ ^13^ ^]^ In addition, activating the A3 or A2b receptor can improve the erectile function of diabetes mellitus‐related ED (DMED) rats.^[^ ^14^, ^15^ ^]^ These studies indicate that the adenosine signaling pathway may be a potential target in penile rehabilitation. However, the specific adenosine receptor subtype mediating adenosine‐induced erection and promoting penile rehabilitation remains controversial. The roles of adenosine and adenosine receptors (A1, A2a, A2b, and A3) in penile rehabilitation of refractory ED, such as DMED, age‐related ED (AED), and bilateral cavernous nerve crush (BCNC)‐related ED, have not been clarified. Notably, excess adenosine in penile tissues can cause penile fibrosis.^[^ ^16^ ^]^ Therefore, finding an optimal regimen for activating the adenosine signaling pathway is needed to improve erectile function and avoid penile fibrosis concurrently.

Of note, the half‐life of adenosine is extremely short due to its rapid uptake and degradation, hindering its direct application in clinics.^[^ ^17^ ^]^ Several in vivo and in vitro studies have demonstrated that dipyridamole (DIP), as an adenosine transporter inhibitor, can significantly increase the local adenosine concentration by attenuating adenosine uptake.^[^ ^18^ ^]^ DIP is an inexpensive, safe, readily available drug, which has been used for a long time to prevent strokes and other vascular diseases due to its antiplatelet and vasodilating activities.^[^ ^19^ ^]^ Several studies have found that DIP has antioxidant, neuroprotective, antiapoptotic, and antifibrotic properties, which indicates that DIP has the potential to treat ED.^[^ ^20^, ^21^, ^22^ ^]^ However, there is little information available on the effects of penile rehabilitation and possible mechanisms for DIP, which is addressed in this study.

The present study aimed to determine the effects and mechanisms of adenosine and adenosine receptor subtypes in regulating erectile function and promoting penile rehabilitation. The optimal regimen for adenosine receptor agonists was explored in vitro. In vivo, the therapeutic effects of an oral adenosine transporter inhibitor (DIP) were also investigated.

## 2 Results

### 2.1 Expression of Adenosine Receptors in Single‐Cell Sequencing of Human Penile Cavernous Tissue

**Figure 1:**
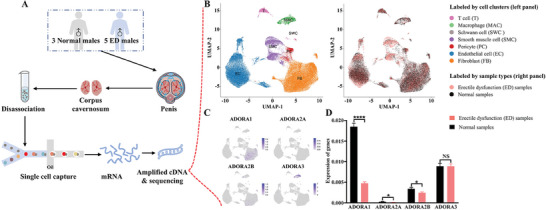
Expression of adenosine receptors in single‐cell sequencing of human penile cavernous tissue. A) Schematic illustration of single‐cell sequencing of human penile cavernous tissue. B) UMAP plot of all corpus cavernosum cells from eight samples (three normal vs five ED patients). Cells are colored according to their clusters (left panel) or sample types (right panel). C) Cell specific expression of adenosine receptors in all corpus cavernosum cells. D) Differential expression of four adenosine receptors in normal and ED patients. Statistical analysis was performed using an unpaired t‐test. NS: not significant. ^*^ *P* < 0.05, ^****^ *P* < 0.0001.

A total of 64,993 cells from five ED patients and three healthy controls were enrolled into analyses after quality control (**Figure** **1**A). In the Uniform Manifold Approximation and Projection (UMAP) plot (Figure 1B), the cells were grouped into seven clusters: endothelial cells (ECs), fibroblasts (FBs), Pericytes (PCs), Smooth muscle cells (SMCs), Schwann cells (SWCs), Macrophages (MACs), and T cells. We further assessed the cell specific expression of adenosine receptors (Figure 1C). It was found that adenosine A1, A2b, and A3 receptor were highly expressed in ECs and FBs; in FBs and MACs; and in FBs, ECs, and MACs, respectively. However, the adenosine A2a receptor was rarely expressed in all the cells, which may be attributed to insufficient sequencing depth. We further determined the expression of adenosine receptors in normal and ED samples (Figure 1D). Significant reductions of adenosine A1, A2a, and A2b receptor levels were identified in ED samples (all *P* < 0.05), while the expression of the adenosine A3 receptor remained unchanged (*P* > 0.05).

### 2.2 The Adenosine A2b Receptor Mediates Adenosine‐Induced Penile Erection

**Figure 2:**
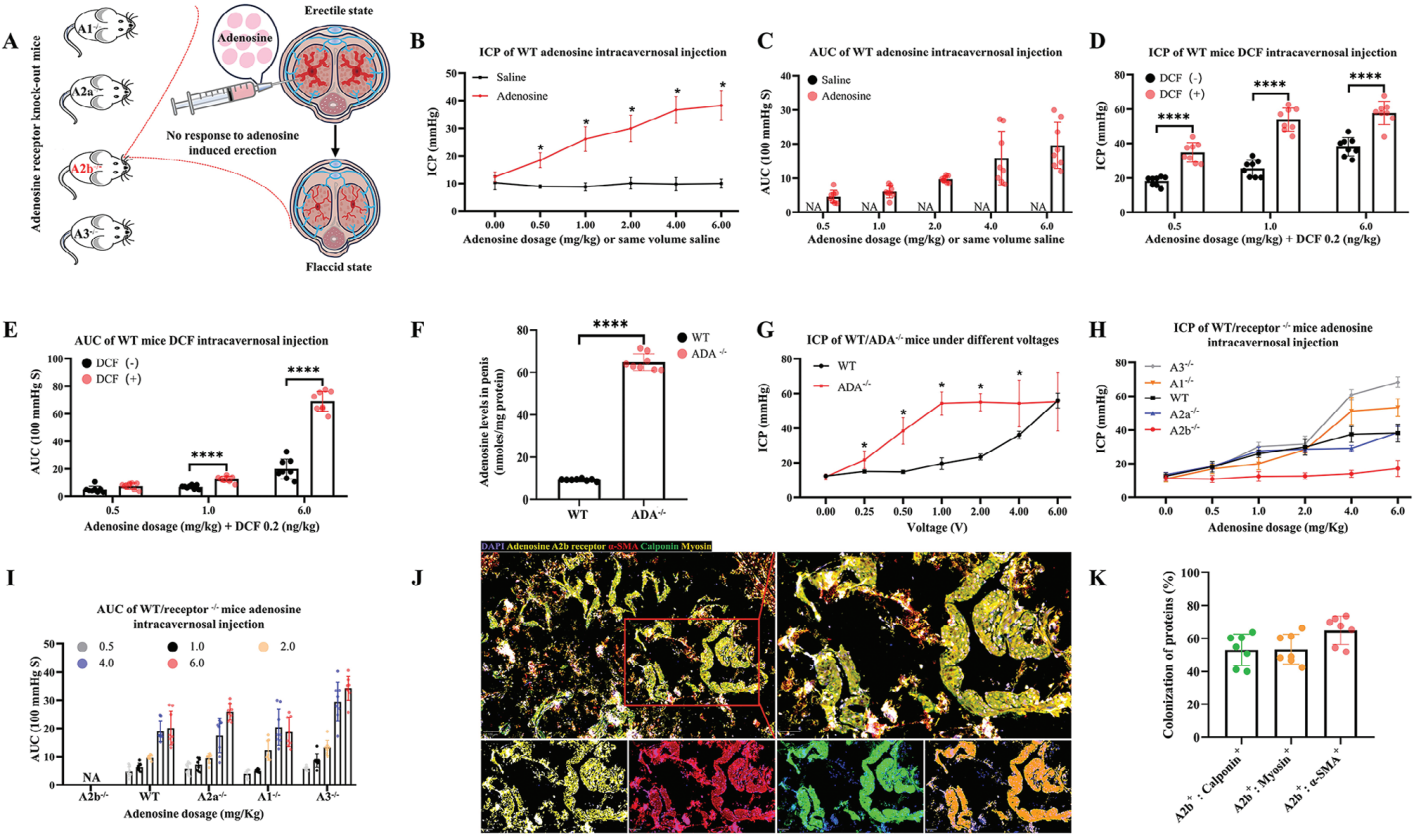
Adenosine A2b receptor mediates adenosine‐induced penile erection. A) Schematic diagram of adenosine intracavernosal injection in adenosine receptors knock‐out mice. B,C) ICPs and AUCs of WT mice under adenosine intracavernosal injection. D,E) ICPs and AUCs of WT mice under adenosine and DCF intracavernosal injection. F) Adenosine levels in the penis of ADA^−/−^ mice. G) ICPs of ADA^−/−^ mice under different voltages. H,I) ICPs and AUCs of adenosine receptor knock‐out (A1^−/−^, A2a^−/−^, A2b^−/−^, A3^−/−^) and WT mice. N = 8 in each group. Statistical analysis was performed using an unpaired t‐test. ^*^ *P* < 0.05, ^****^ *P* < 0.0001. J,K) Colocalization of adenosine A2b receptor, calponin, myosin and α‐SMA in the corpus cavernosum of rats. Scale bars = 100 or 50 µm. N = 7.

To identify the adenosine receptor subtypes responsible for adenosine‐induced erection, adenosine receptors knock‐out mice were constructed and then challenged with adenosine (**Figure** **2**A). In Figure 2B,C, adenosine intracavernosal injection in wild‐type (WT) mice induced significant increases of ICPs and area under curves (AUCs), indicating increased erectile function. Given the core role of adenosine deaminase (ADA) in adenosine degradation, we used deoxycoformycin (DCF, an irreversible ADA inhibitor) to elevate adenosine levels in the penis. As shown in Figure 2D,E, the DCF‐treated mice had higher ICPs and AUCs than the saline control group (all *P* <0.0001). Besides, ADA knock‐out (ADA^−/−^) mice were further used to verify the results. In Figure 2F and Figure S1 (Supporting Information), the ADA^−/−^ mice displayed higher adenosine levels than the WT mice (64.78 ± 3.95 vs 9.25 ± 0.49, *P* < 0.0001). Accordingly, in Figure 2G, the ICPs of ADA^−/−^ mice were higher than the WT mice under the voltage of 0.25–4 volts (*P* < 0.05). These data suggested that adenosine could improve erectile function, but the downstream adenosine receptors that mediate this effect have not been identified. Therefore, adenosine receptors knock‐out mice (A1^−/−^, A2a^−/−^, A2b^−/−^, and A3^−/−^) were treated with different adenosine dosages to identify the responsible receptors. The ICPs and AUCs of the A2b^−/−^ mice were lower than all other groups (all *P* <0.05) and remained unchanged when the mice were treated with adenosine (Figure 2H–I). These data suggested that adenosine‐induced erection was mediated by the A2b receptor rather than by the A1, A2a or A3 receptors. Immunofluorescence showed that the expression of the adenosine A2b receptor mainly overlapped with the expression of calponin, myosin, and α‐SMA (Figure 2J). The average proportions were 53.05% for A2b^+^‐Calponin^+^ cells, 53.42% for A2b^+^‐Myosin^+^ cells, and 64.87% for A2b^+^‐α‐SMA^+^ cells in all cells, respectively (Figure 2K). Additionally, F4/80 (a marker of macrophage), was rarely expressed in the cavernosum (Figure S2, Supporting Information), and overlapped with A2b (Figure S3, Supporting Information). CD31^+^ (a marker of endothelial cells) cells differed in spatial location from A2b^+^ cells (Figure S4, Supporting Information). Given the above results and physiological rationality, we selected smooth muscle cells for further experiments.

### 2.3 Under Normoxia, Adenosine A2a Receptor Activation Up‐Regulates HIF‐1α and Down‐Regulates PDE5

**Figure 3:**
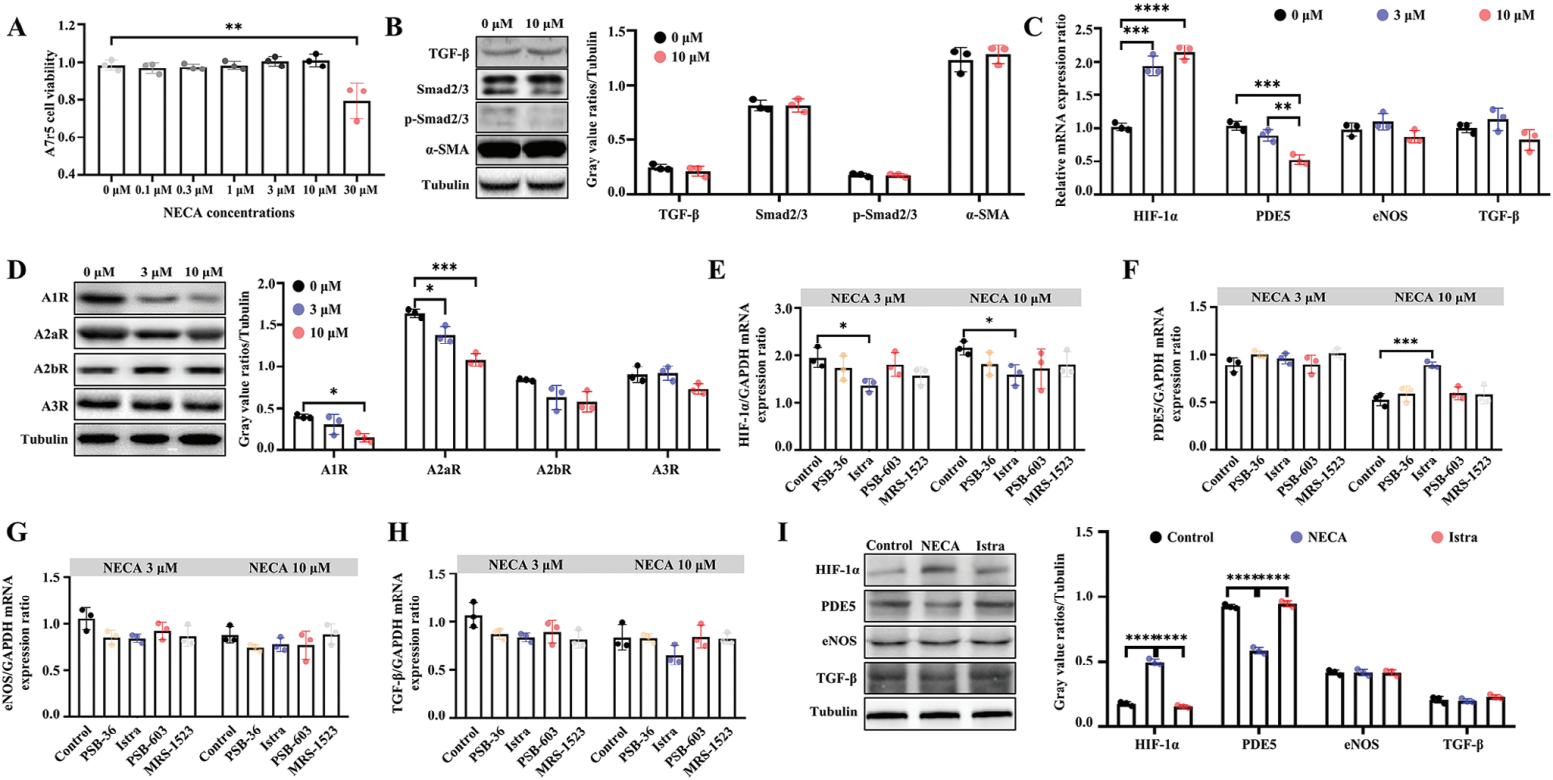
Under normoxic condition, adenosine A2a receptor activation up‐regulates HIF‐1α, and down‐regulates PDE5. A) A7r5 cell viability under NECA stimulation. The cell viability was detected by the CCK‐8 test. B) Representative WB protein bands and statistical analysis of TGF‐β, Smad2/3, p‐Smad2/3, α‐SMA, and Tubulin. C) Relative mRNA expressions of HIF‐1α, PDE5, eNOS, and TGF‐β. D) Representative WB protein bands and statistical analysis of A1, A2a, A2b, and A3 receptors under NECA stimulation. E–H) Relative mRNA expressions of HIF‐1α, PDE5, eNOS, and TGF‐β were analyzed. The A7r5 cells were treated by NECA (3 and 10 µm) and adenosine receptor inhibitors (PSB‐36 for the A1 receptor, Istra for the A2a receptor, PSB‐603 for the A2b receptor, and MRS‐1523 for the A3 receptor). I) Representative WB protein bands and statistical analysis of HIF‐1α, PDE5, eNOS, TGF‐β, and Tubulin. The A7r5 cells were treated by NECA (10 µm) and A2a receptor inhibitors (Istra). Statistical analysis was performed using an unpaired t‐test or ANOVA. N = 3. ^*^ *P* < 0.05, ^**^ *P* < 0.01, ^***^ *P* < 0.001, ^****^ *P* < 0.0001.

Overactivation of the adenosine pathway can lead to penile fibrosis. To find the optimal regimen for promoting penile rehabilitation and avoiding penile fibrosis, seven different concentrations of 5′‐N‐Ethylcarboxamidoadenosine (NECA, a non‐selective high affinity adenosine receptor agonist) were used to treat A7r5 cells (0–30 µm). CCK‐8 tests showed that NECA (30 µm) could suppress cell growth and activity (*P* < 0.01), while 0–10 µm could not (**Figure** **3**A). Moreover, NECA concentrations of 10 and 30 µm did not activate the TGF‐β/Smad pathway, indicating less likelihoods of inducing fibrosis (Figure 3B; Figure S5, Supporting Information). The protein expression of TGF‐β, Smad2/3, p‐Smad2/3, and α‐SMA was not altered in A7r5 cells (all *P* > 0.05). Therefore, we selected concentrations of 3 and 10 µm for further exploration. After stimulation with NECA (3 or 10 µm), the mRNA expression of HIF‐1α increased (*P* < 0.001), while that of PDE5 decreased (*P* < 0.01). The mRNA levels of eNOS and TGF‐β remained unchanged (all *P* > 0.05, Figure 3C; Figure S6, Supporting Information). Western blot (WB) revealed that A1 and A2a receptor levels were significantly decreased compared to the control group (*P* < 0.05, Figure 3D). To find the corresponding adenosine receptor that mediates this effect, four different inhibitors for A1 (PSB‐36), A2a (Istra), A2b (PSB‐603), and A3 (MRS‐1523) were used to treat A7r5 cells. After stimulation with NECA (3 and 10 µm), the A2a receptor inhibitor (Istra) could reverse the up‐regulation of HIF‐1α mRNA and protein (*P* < 0.05), while the other inhibitors did not (*P* > 0.05, Figure 3E,I). Istra could reverse the down‐regulation of PDE5 mRNA and protein (*P* < 0.001, Figure 3F,I). In Figure 3G–I, inhibiting all four adenosine receptors did not alter the mRNA or protein expression of eNOS and TGF‐β (*P* > 0.05). These data suggest that the A2a receptor, rather than the A2b receptor, is responsible for regulating HIF‐1α and PDE5 under normoxia.

### 2.4 Under Hypoxia, Adenosine A2b Receptor Activation Up‐Regulates HIF‐1α and Down‐Regulates PDE5

**Figure 4:**
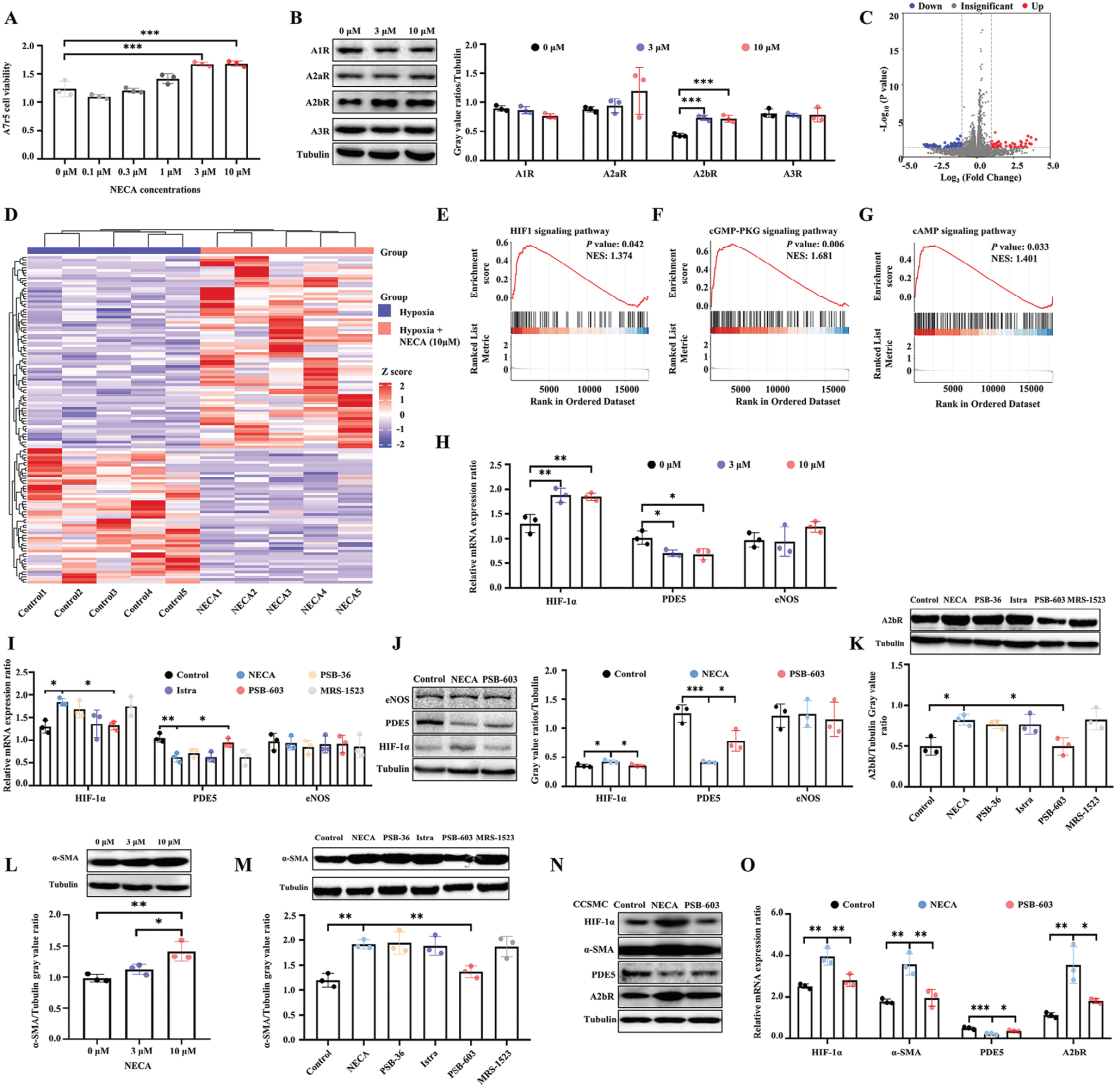
Under hypoxia, adenosine A2b receptor activation up‐regulates HIF‐1α, and down‐regulates PDE5. A) A7r5 cell viability under NECA stimulation. B) Representative WB protein bands and statistical analysis of A1, A2a, A2b, and A3 receptors. C) Volcano plot visualizing the altered gene expression profiles under the stimulation of NECA. D) Cluster and heatmap of the differentially expressed genes. E–G) HIF‐1, cGMP‐PKG, and cAMP signaling pathways were significantly enriched by GSEA. H) Relative mRNA expressions of HIF‐1α, PDE5, and eNOS. I) Relative mRNA expressions of HIF‐1α, PDE5, and eNOS. The A7r5 cells were treated with NECA (10 µm) and adenosine receptor inhibitors. J) Representative WB protein bands and statistical analysis of HIF‐1α, PDE5, and eNOS. The A7r5 cells were treated by NECA (10 µm) and A2b receptor inhibitor (PSB‐603). K) Representative WB protein bands and statistical analysis of A2b receptor. The A7r5 cells were treated with adenosine receptor inhibitors. L) Representative WB protein bands and statistical analysis of α‐SMA. M): Representative WB protein bands and statistical analysis of α‐SMA. N) Representative WB protein bands and statistical analysis of HIF‐1α, α‐SMA, PDE5, and A2b receptor. The primary corpus cavernosum smooth muscle cells were treated by NECA (10 µm) and A2b receptor inhibitor (PSB‐603). O) Relative mRNA expressions of HIF‐1α, eNOS, PDE5, and A2b receptor. Statistical analysis was performed using an unpaired t‐test or ANOVA. N = 3 or 5. ^*^ *P* < 0.05, ^**^ *P* < 0.01, ^***^ *P* < 0.001.

The accumulation of adenosine is higher under hypoxia (micromole) than under physiological conditions (nanomole).^[^ ^23^, ^24^ ^]^ To evaluate the function of adenosine under hypoxia, A7r5 cells were cultured in 1% O_2_. The CCK‐8 test found that NECA (3 and 10 µm) promoted cell growth and activity under hypoxia (*P* <0.001), while other concentrations (0.1–1 µm) did not (*P* >0.05, **Figure** **4**A). Further WB revealed that NECA increased the expression of the A2b receptor instead of the A1, A2a, or A3 receptors (*P* <0.001, Figure 4B). To explore the potential pathways responsible for NECA stimulation, cells were subjected to RNA sequencing. After stimulation with NECA (10 µm), the gene expression profiles were significantly altered, of which 52 genes were up‐regulated and 74 genes were down‐regulated (Figure 4C; Figure S7, Supporting Information). The normalized counts of these differentially expressed genes are visualized in Figure 4D. Further gene set enrichment analysis (GSEA) showed that the HIF‐1, cGMP‐PKG, and cAMP signaling pathways were significantly enriched (Figure 4E–G). The downstream pathways related to HIF‐1 signaling pathways, such as the VEGF signaling pathway, lysosome, efferocytosis, propanoate, and ether lipid metabolism, were also enriched (Figure S8A–E; Table S1, Supporting Information). Similar findings were replicated by single‐cell sequencing (Figure S9A–F, Supporting Information). These data indicate that NECA stimulation may activate the HIF‐1α and PDE5 pathways, which mediate adenosine‐induced erection.

In line with the sequencing results, polymerase chain reaction showed that stimulation with NECA (3 and 10 µm) could also elevate HIF‐1α and downstream molecules, and decrease PDE5 (*P* <0.05); however, the mRNA levels of HIF‐2α and eNOS remained unchanged (*P* > 0.05, Figure 4H; Figure S10A–E, Supporting Information). To confirm the role of the A2b receptor in regulating HIF‐1α and PDE5, four selective adenosine receptor inhibitors for the A1, A2a, A2b, and A3 receptors were used to treat A7r5 cells under hypoxia and NECA stimulation. Figure 4I discloses that only the A2b receptor inhibitor (PSB‐603) reversed the increase of HIF‐1α and the decline of PDE5 (*P* < 0.05), in line with the alteration of PDE5 and HIF‐1α proteins (*P* < 0.05, Figure 4J). WB validated that only PSB‐603 could lower the expression of the A2b receptor protein (*P* <0.05), while the other selective inhibitors could not (*P* > 0.05, Figure 4K). α‐SMA is an indicator of the contractile ability and contractile phenotype of the smooth muscle cells. NECA (10 µm) significantly increased the expression of α‐SMA (*P* < 0.05, Figure 4L). The elevated α‐SMA protein level could be down‐regulated only by the A2b receptor inhibitor PSB‐603 but not by other selective adenosine receptor inhibitors (*P* < 0.01, Figure 4M), indicating the mediating role of the A2b receptor under hypoxia.

To further verify these findings, we also extracted and cultured primary corpus cavernosum smooth muscle cells (CCSMC) from the rat penis. Similar to A7r5 cells, in CCSMC, stimulation with NECA (10 µm) increased the expression of the HIF‐1α, α‐SMA, and A2b receptor proteins and decreased the expression of the PDE5 protein (Figure 4N). The inhibition of the A2b receptor canceled out the effects. The same trends were replicated in their corresponding mRNAs (Figure 4O).

### 2.5 Activating the A2b Receptor Promotes Penile Rehabilitation in three Refractory ED Models

**Figure 5:**
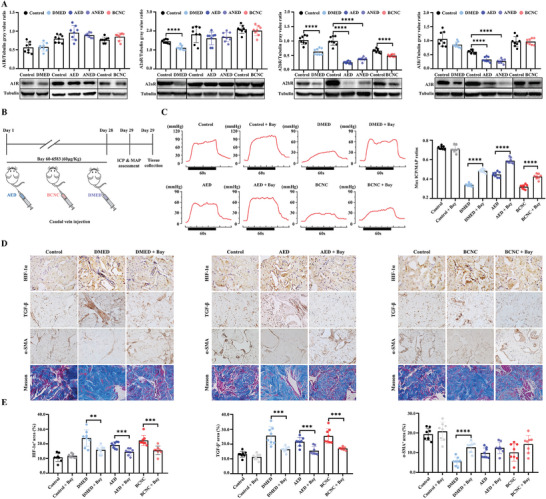
Activating the A2b receptor promotes penile rehabilitation in three rat models of refractory ED. A) Representative WB protein bands and statistical analysis of A1, A2a, A2b, and A3 receptors in AED, BCNC, and DMED rats. B) Study protocol of Bay 60–6583 treatment on AED, BCNC, and DMED rats. C) Representative traces and statistical results of ICP in each group after Bay 60–6583 treatment. D,E) Representative Masson staining, IHC images, and statistical analysis of HIF‐1α, TGF‐β, and α‐SMA in each group; Original magnification × 200. N = 8. Statistical analysis was performed using Student‐t test or ANOVA. ^**^ *P* < 0.01, ^***^ *P* < 0.001, ^****^ *P* < 0.0001.

We further evaluated the expression of the A2b receptor in three refractory ED rat models. Representative WB images are shown in **Figure** **5**A. For the A1 receptor, no differences were detected among the control, DMED, AED, aged but no ED (ANED), and BCNC groups (all *P* > 0.05). In Figure 5A, the A2a receptor was down‐regulated in the DMED group (*P* <0.0001) but not in the AED, ANED, or BCNC groups (all *P* > 0.05). For the A2b receptor, a significant reduction was noted in all three refractory ED models (all *P* < 0.0001). Decreased expression of the A3 receptor was observed in the AED and ANED groups (*P* <0.0001) but not in the DMED or BCNC group (*P* > 0.05). The immunohistochemistry (IHC) images displayed the same results as the WB images (Figure S11, Supporting Information). To further explore the effect of activating the A2b receptor in refractory ED models, a selective A2b receptor agonist (Bay‐606583) was used to treat refractory ED rats (Figure 5B). Treatment with Bay‐606583 improved the erectile function of DMED, AED, and BCNC rats (Figure 5C). The maximum ICP/mean arterial blood pressure (MAP) ratios of the treated groups were significantly higher than that of the DMED group (0.48 ± 0.02 vs 0.34 ± 0.04, *P* < 0.0001), AED group (0.59 ± 0.03 vs 0.45 ± 0.03, *P* < 0.0001), and BCNC group (0.42 ± 0.03 vs 0.32 ± 0.02, *P* < 0.0001). These data support the therapeutic effects of activating the A2b receptor in refractory ED models.

The critical molecules of penile rehabilitation were assessed. In Figure S12 (Supporting Information), no difference in adenosine levels was detected between the control group and the three ED models (*P* > 0.05). Treatment with Bay‐606583 significantly elevated the cAMP concentration in the control (53.93 ± 4.74 vs 39.51 ± 3.56, *P* <0.05), DMED (52.35 ± 4.35 vs 34.57 ± 3.56, *P* < 0.05), AED (46.02 ± 2.57 vs 32.40 ± 2.96, *P* < 0.05), and BCNC (45.04 ± 3.16vs 33.98 ± 3.75, *P* < 0.05, Figure S13, Supporting Information) groups. The expressions of HIF‐1α, TGF‐β, and α‐SMA were detected using IHC (Figure 5D). In the control group, Bay‐606583 had no effect on the expression of HIF‐1α, TGF‐β, or α‐SMA (all *P* > 0.05). However, in the DMED group, Bay‐606583 decreased the expression of HIF‐1α (0.24 ± 0.06 vs 0.16 ± 0.03, *P* < 0.01) and TGF‐β (0.26 ± 0.06 vs 0.16 ± 0.02, *P* <0.001) and increased the expression of α‐SMA (0.06 ± 0.02 vs 0.13 ± 0.03, *P* < 0.0001, Figure 5E). HIF‐1α and TGF‐β were also lowered in the AED and BCNC groups after treatment (all *P* <0.001). Notably, Bay‐606583 treatment did not increase the expression of α‐SMA in the AED group (0.10 ± 0.03 vs 0.13 ± 0.03, *P* > 0.05) or the BCNC group (0.10 ± 0.04 vs 0.15 ± 0.03, *P* > 0.05).

### 2.6 Effects of DIP on ED in BCNC Rats

**Figure 6:**
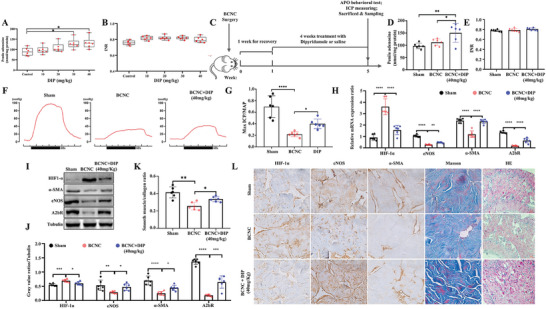
DIP treatment promotes penile rehabilitation of BCNC rats. A,B) The significantly increased adenosine levels were observed following the administration of DIP 30 and 40 mg kg^−1^ to the adult rats, and no obvious difference in INR was found among different DIP groups. C): Study protocol of DIP treatment on BCNC rats. D,E) There was a significant increase in the adenosine levels of penile tissues after oral administration of DIP at a dose of 40 mg kg^−1^ to BCNC rats and no significant difference in the mean INR value. F,G) Representative traces of ICP, and statistical analysis of max ICP/MAP in groups. H) Relative mRNA expressions of HIF‐1α, α‐SMA, eNOS, and A2bR in groups. I,J) Representative WB protein bands and statistical analysis of HIF‐1α, α‐SMA, eNOS, and A2bR in groups. K) Penile smooth muscle/collagen ratio in groups. L) Representative H&E staining, Masson's staining and immunochemistry images of eNOS, HIF‐1α, and α‐SMA in each group. Original magnification × 200. Statistical analysis was performed using ANOVA. N = 6. ^*^ *P* < 0.05, ^**^ *P* < 0.01, ^***^ *P* < 0.001, ^****^ *P* < 0.0001.

DIP is an adenosine transporter inhibitor that can significantly increase the local adenosine concentration. We further assessed the therapeutic effects and mechanisms of DIP in BCNC rats. After treatment, the DIP 40 mg Kg^−1^ group had a higher penile adenosine level than the control group (89.50 ± 17.60 vs 131.84 ± 25.09, *P* < 0.05, **Figure** **6**A). Given the anticoagulative property of DIP, we also assessed the international normalized ratio (INR) values of the five groups, detecting no difference (*P* > 0.05, Figure 6B). Considering that the penile adenosine concentration was highest in the DIP 40 mg Kg^−1^ group, the BCNC rats were then treated with DIP (40 mg Kg^−1^) for four weeks, as displayed in Figure 6C. Treatment with DIP (40 mg/Kg) significantly increased penile adenosine levels compared with those in the control group (150.50 ± 34.54 vs 97.42 ± 10.30, *P* < 0.01, Figure 6D), with no impact on the INR (*P* > 0.05, Figure 6E).

The erectile function of the BCNC group was improved after DIP treatment, as indicated by a higher max ICP/MAP ratio and AUC (all *P* < 0.05, Figure 6F–G; Figure S14, Supporting Information). Further tests showed that DIP treatment decreased the mRNA expression of HIF‐1α (*P* < 0.05) and increased the mRNA expression of eNOS, α‐SMA, and the A2b receptor (*P* < 0.0001, Figure 6H), in line with the protein alteration (Figure 6I,J). H&E staining was performed to study the microscopic structure of the penis, and Masson's trichrome staining was performed to identify the penile smooth muscle/collagen ratio. The percentage of smooth muscle/collagen tissue within the rat corpora cavernosa was 5.95% ± 1.25% in the sham group, 3.55% ± 1.12% in the BCNC group, and 4.76% ± 0.68% in the DIP therapy group (*P* < 0.05, Figure 6K,L). IHC was performed to verify the expression of HIF‐1α, eNOS, and α‐SMA in the corpus cavernosum (Figure 6L). Compared with those in the control group, the oral administration of DIP at a dose of 40 mg kg^−1^ partially reversed the expression levels of HIF‐1α, eNOS, α‐SMA, and A2b in BCNC rats (all *P* < 0.05).

## 3 Discussion

The therapeutic effects of adenosine and its mechanisms on refractory ED have been less investigated in previous studies. Using single‐cell RNA sequencing technology, we found that the adenosine A1, A2a, and A2b receptor levels in ED patients were significantly reduced. In vivo, A2b receptor knock‐out abolished adenosine‐induced dramatic increase in the ICP and AUC instead of A1, A2a, and A3. In addition, our study indicated that under hypoxic and normoxic conditions, the A2b and A2a receptors were activated, respectively. Activating the A2b receptor promoted penile rehabilitation of three refractory ED rat models (DMED, AED, and BCNC) by alleviating hypoxia and fibrosis. Given the extremely short half‐life of adenosine, an oral drug, DIP, is considered to regulate adenosine concentration in animal models. DIP had significant therapeutic effects on BCNC rats by attenuating hypoxia and fibrosis.

It has been well documented that adenosine injection in the cavernosum can induce penile erection.^[^ ^7^, ^25^ ^]^ However, the receptors responsible for these effects have been controversial in previous studies. Chiang PH et al. found that adenosine could induce the relaxation of cavernosal strips from rabbits via the A2 receptor. A selective A2a receptor inhibitor (CGS 21680) could not abolish the relaxing effect.^[^ ^26^ ^]^ It was postulated that the A2b receptor might be the responsible adenosine receptor. Shreds of evidence from Kataoka K and his colleagues supported their results.^[^ ^11^ ^]^ It was reported that the A2b antagonist, rather than the A2a antagonist, could suppress adenosine‐induced relaxation of cavernosal strips from rabbits, which was in line with Ning C's study.^[^ ^12^ ^]^ These studies indicated that the A2b receptor mediates the pro‐erectile effect of adenosine. However, in contrast to the above‐mentioned findings, Noto T et al. reported that a selective A2a receptor inhibitor (ZM241385) could abolish the erection induced by cavernosal injection of adenosine in dogs.^[^ ^13^ ^]^ In mice, Tostes RC et al. reported that adenosine could relax cavernosal strips by activating the A2a or A2b receptor.^[^ ^9^ ^]^ Validation in human cavernosal strips also supported that adenosine can induce penile relaxation by activating sensitive A2a and insensitive A2b receptors.^[^ ^10^ ^]^ The relaxing effect of activating the A2b receptor is more evident than that of activating the A2a receptor. These studies highlighted that both the A2a and A2b receptors could mediate the pro‐erectile effect of adenosine. Among our adenosine receptors knock‐out mice, A2b^−/−^ mice displayed no erection, and A2a ^‐/−^ mice had a normal erection similar to that of WT mice, indicating that A2b is the responsible receptor rather than A2a. Previous studies have been performed mainly in animals using adenosine receptor antagonists or inhibitors, while our study was performed based on human corpus cavernosum tissues and adenosine receptor knock‐out mice, which may be more convincing.

During hypoxia or ischemia, adenosine can play a protective role by reducing cell apoptosis, promoting cell proliferation, and suppressing inflammation.^[^ ^27^ ^]^ However, long‐term and excess adenosine can also induce apoptosis, hypoxia, and tissue fibrosis.^[^ ^16^, ^28^ ^]^ The effects of activating the adenosine signaling pathway are bidirectional and dependent on the adenosine concentration. The physiological extracellular concentration of adenosine is estimated to be 30–200 nm.^[^ ^29^ ^]^ During hypoxia, ischemia, or inflammation, adenosine levels can be significantly elevated to the micromolar level, inducing pathological alterations. Wen's study showed that NECA (20 µm) could induce the proliferation of corpus cavernosum fibroblasts.^[^ ^16^ ^]^ Our study showed that 3–10 µm NECA is a safe concentration for maintaining cell activity under hypoxia. In addition, 30 µm NECA activated the TGF‐β/Smad pathway in fibrosis. Thus, there is an appropriate range of local adenosine concentrations in the penis, which can enhance cell activity and avoid triggering fibrosis concurrently. Future drug development targeting adenosine receptors in the penis should take this into account. Of note, the responsible adenosine receptors are A2a under normoxic conditions and A2b under hypoxic conditions. This difference may be explained by the affinity of different adenosine receptors.^[^ ^17^ ^]^ Both G_s_‐coupled A2a and A2b receptors can activate adenylate cyclase and trigger cAMP‐dependent downstream signaling events. Under physiological conditions with low adenosine concentrations, the A2a receptor, which has high affinity, mediates the effect of adenosine. However, the A2b receptor is a low‐affinity receptor, whose expression is significantly elevated under hypoxia.^[^ ^30^ ^]^ A2a is replaced by the significantly increased A2b receptor during hypoxia.

At present, PDE5i, which acts on the nitric oxide‐cyclic guanosine monophosphate (NO‐cGMP) signaling pathway, is the preferred therapy for ED. Although PDE5i provides safe and effective treatment for ED, they have several drawbacks and limitations. The effectiveness of PDE5i reaches 60–70%, but 30–35% of patients fail to respond to a PDE5i.^[^ ^2^, ^3^ ^]^ Patients who have diabetes and severe neurological damage in particular exhibit a poor response to PDE5i.^[^ ^31^ ^]^ In addition to PDE5i, our study supports that activating the A2b receptor can promote penile rehabilitation of refractory ED. The mechanism of penile erection and rehabilitation induced by adenosine is different from that induced by PDE5i. PDE5i inhibits PDE5 and accordingly up‐regulates cGMP. Increased cGMP can down‐regulate the calcium concentration, relax smooth muscle cells, and finally induce erection.^[^ ^32^ ^]^ However, adenosine can induce prolonged erection not only through the NO‐cGMP pathway but also through the cAMP‐protein kinase A (PKA) pathway.^[^ ^6^ ^]^ In clinical settings, this signaling pathway may be an attractive therapeutic target for the treatment of refractory ED. However, given the wide distribution of adenosine receptors, there is a long way to go from bench to bedside.

DIP is a well‐known adenosine reuptake inhibitor that can block adenosine transporters on the cell membrane, leading to elevated extracellular adenosine concentrations.^[^ ^21^ ^]^ Therefore, we hypothesize that DIP inhibits cellular adenosine uptake, increases the extracellular adenosine concentration, and enhances the recovery of erectile function in BCNC rats. Previous studies have shown that intra‐abdominal administration of DIP has anti‐apoptotic and anti‐fibrotic effects but has failed to improve erectile function in a rat model of cavernous nerve crush injury.^[^ ^33^ ^]^ Nevertheless, it appears worthwhile to reconsider whether 10 mg kg^−1^ day^−1^ DIP is ineffective at improving erectile function, given the insufficient dosage.^[^ ^34^ ^]^ Clinically, DIP was administered initially at a dose of 100 mg day^−1^ and was increased to a maximal dose of 400 mg day^−1^ if necessary.^[^ ^18^ ^]^ According to the dose conversion between humans and rats (human: rat = 1: 6.17), the dose of DIP in rats in the present study was equivalent to the DIP in a 60 kg human (calculated as follows: 41.13 mg kg^−1^ day^−1^ = 6.17 × 400 mg 60 kg^−1^). Considering the safety of this drug and its potential in the treatment of refractory ED, further clinical trials are necessary.

Some limitations of this study should be recognized. First, the optimal regimen of NECA was determined in vitro, rather than in vivo, which is inequivalent to the extracellular adenosine in the body. Besides, the increase of extracellular adenosine was limited (89.5 vs 131.8 nmol mg^−1^ protein) after a four‐week treatment with DIP (40 mg kg^−1^ day^−1^). Higher dosages and more extended periods of treatment may bring more benefits. Therefore, the optimal regimen in vivo still needs further exploration. Moreover, the effects of activating the A2a receptor in normal rats and DMED rats were not explored in this study. It is potential to improve erectile function in healthy males and DMED deserves further exploration. Additionally, the single‐cell sequencing results should be further validated in males, which will be addressed in our future research.

In summary, on the basis of single‐cell sequencing technology and adenosine receptors knock‐out mice, our results reveal that the A2b receptor mediates adenosine‐induced erection. Activating the A2b receptor promotes penile rehabilitation of refractory ED by alleviating hypoxia and fibrosis. An oral drug, DIP, displays significant therapeutic effects on refractory ED by attenuating hypoxia and fibrosis. The drug is non‐invasive for refractory ED patients, showing therapeutic potential in clinics.

## 4 Experimental Section

### Experimental Design

This study included the following steps: I) In human corpus cavernosum, assessing the expressions of adenosine receptors (A1, A2a, A2b, and A3) by single‐cell RNA sequencing in three healthy controls and 5 ED patients; II) In mice and rats, determining the effects of adenosine and responsible adenosine receptor subtypes in regulating erectile function; III) In A7r5 (smooth muscle cells of the rat thoracic artery) and primary CCSMC, exploring the optimal concentration and mechanism of adenosine in regulating erectile function under normoxic and hypoxic conditions; IV) In rats, determining the therapeutic effects of activating the adenosine A2b receptor in three refractory ED models (AED, BCNC, and DMED); V) In rats, finding a safe and effective dose of DIP (an adenosine transporter inhibitor), and further assessing the efficacy and mechanisms of DIP in BCNC rats.

### Single‐Cell Sequencing‐Based Analysis of Adenosine Receptor Expression in the Human Corpus Cavernosum

Human corpus cavernosum tissues were collected from eight males by Zhao et al.^[^ ^35^ ^]^ Of them, three were from healthy controls, two were from diabetic ED patients and three were from non‐diabetic ED patients. The human samples were collected under the guidance of the Ethics Committee of Shanghai General Hospital (License No. 2021SQ259) and following the criteria established by the Declaration of Helsinki. Informed consent was obtained from all subjects in the original study. All the experiments were performed in accordance with the relevant guidelines and regulations. The penile tissues were prepared and sequenced using 10 × Genomics with an Illumina NovaSeq 6000 (San Diego, CA, USA). The quality control process and parameters were detailed in the original study.^[^ ^35^ ^]^ The top 20 principal components were used to cluster and further perform UMAP dimensionality reduction. The marker genes of each cluster were in line with those of Zhao's study.^[^ ^35^ ^]^ The expression differences of adenosine A1, A2a, A2b, and A3 receptors were visualized using the FeaturePlot function in Seurat package after Z‐score standardization and tested by t tests using the ggpubr package.

### Animal Groups and Cellular Intervention

WT, ADA knock‐out, and adenosine A2b receptor knock‐out mice were generated at the University of Texas Medical School in Houston, USA. A1^–/–^ mice were from J. Schnermann (NIDDK, NIH, Bethesda, MD, USA); A2aR^–/–^ mice were from J.‐F. Chen (Boston University School of Medicine, Boston, MA, USA) and A3^–/–^ mice were from M. Jacobson (Merck Research Laboratories, West Point, PA, USA). The ADA^−/−^ mice had a mixed background of the 129/sV, C57BL/6, and FVB/N strains. Besides, all adenosine receptor knock‐out mice were bred on a C57BL/6 background. All male Sprague‐Dawley rats were purchased from Dashuo Experimental Animal Co. Ltd. (Chengdu, Sichuan Province, China). All the experiments were performed in accordance with the relevant guidelines and regulations. All the mice and rats were housed and cared for under strict guidelines, and this study was approved by the Animal Care and Use Committee at the University of Texas Health Science Center and Animal Ethics Committee of West China Hospital, Sichuan University.

*For series II*: five experiments involving 48 WT mice, 8 ADA^−/−^ mice, 8 A1^−/−^ mice, 8 A2a^−/−^ mice, 8 A2b^−/−^ mice, 8 A3^−/−^ mice and 7 rats were carried out. In the first experiment, 16 WT mice were subjected to adenosine intracavernosal injection (0, 0.5, 1.0, 2.0, 4.0, or 6.0 mg Kg^−1^) or corresponding sterile saline injection. In the second experiment, 16 WT mice were equally divided into two groups (injected with DCF (0.2 mg Kg^−1^) vs sterile saline). DCF is an irreversible ADA inhibitor obtained from MedChem Express (Monmouth, NJ, USA). After the injection of DCF or saline, the mice were stimulated by adenosine intracavernosal injection (0.5, 1.0, or 6.0 mg Kg^−1^). Adenosine was obtained from MedChem Express. The third experiment used 8 WT mice and 8 ADA^−/−^ mice to assess the adenosine levels in the penis and erectile function. The fourth experiment used 8 WT mice, 8 A1^−/−^ mice, 8 A2a^−/−^ mice, 8 A2b^−/−^ mice, and 8 A3^−/−^ mice. The mice were injected with six different adenosine dosages (0, 0.5, 1.0, 2.0, 4.0, and 6.0 mg Kg^−1^) in the cavernosum. The fifth experiment used seven rats to investigate the corresponding cell types mediating the adenosine‐induced erection by immunofluorescence staining. Erectile function was evaluated using the maximum ICP and AUC according to previous methods.^[^ ^36^ ^]^ The adenosine levels in the penis were measured using high‐performance liquid chromatography (HPLC).

*For series III*: A7r5 cells (Cell Bank/Stem Cell Bank of the Chinese Academy of Sciences, Beijing, China) were cultured in high‐glucose Dulbecco's modified Eagle's medium (DMEM) at 37 °C in 5% CO_2_. NECA was added to the cells at seven different concentrations (0, 0.1, 0.3, 1, 3, 10, and 30 µmol L^−1^). Cell activity was assessed using a cell counting kit‐8 (CCK‐8, Solarbio, Beijing, China). To further find the responsible adenosine receptor subtypes, A7r5 cells were treated with selective adenosine receptor inhibitors (PSB‐36 for the A1 receptor, Istradefylline (Istra) for the A2a receptor, PSB‐603 for the A2b receptor, and MRS‐1523 for the A3 receptor) under NECA stimulation. NECA and all the other inhibitors were purchased from ApexBIO (Houston, TX, USA). Under hypoxia (1% O_2_ in an incubator), selective adenosine receptor inhibitors were used to treat A7r5 cells, which was further verified in CCSMCs. In addition, RNA‐sequencing was used to explore the enriched signaling pathway under hypoxia and the stimulation of NECA (10 µm). The control group included five samples cultured under hypoxia (1% O_2_ for 24 h). The NECA group included five samples cultured under hypoxia (1% O_2_ for 24 h) and NECA (10 µm).

*For series IV*: Two experiments were performed in this series. The first experiment investigated the expression of adenosine receptors using 56 Sprague‐Dawley male rats, with eight rats in each group. The expression of the A1, A2a, A2b, and A3 receptors was detected by WB and IHC. The adenosine levels were measured via HPLC. The second experiment further evaluated the effect of activating the A2b receptor in treating the three refractory ED models. A selective A2b receptor agonist (Bay 60–6583, R&D Systems Company) was used. The dosage of Bay 60–6583 was 60 µg Kg^−1^ and the solution was continuously injected through the caudal vein for 28 days.

*For series V*: The safe and effective dose of DIP in rats were first explored. Thirty Sprague‐Dawley rats (200 – 220 g) were randomly assigned to five groups (0.9% NaCl control group, 10 mg kg^−1^ group, 20 mg kg^−1^ group, 30 mg kg^−1^ group, and 40 mg kg^−1^ group). The total treatment time was 4 weeks. After 4‐weeks of treatment, whole blood was obtained from the inferior vena cava following anesthesia with 2% isoflurane. The blood sample was centrifuged at 4000 g for 5 min to separate the supernatants, after which the INR was measured using an automated coagulation analyzer (SK5002, Sinothinker Company, Shenzhen, Guangdong Province, China). Then, the efficacy and mechanisms of DIP in the BCNC model was further assessed. Eighteen Sprague‐Dawley rats (200 – 220 g) were randomly assigned to three groups: 1) the sham group: rats underwent the same surgical procedure without nerve crushing; 2) the BCNC group: rats underwent the surgical procedure of BCNC, with orally given vehicle (0.9% NaCl) beginning on the seventh day after surgery without DIP therapy; and 3) the BCNC + DIP 40 mg kg^−1^ group: rats underwent the surgical procedure of BCNC, with the orally given 40 mg kg^−1^ d^−1^ DIP beginning at the seventh day after surgery. The total treatment time was four weeks.

### RNA Sequencing and Enrichment Analysis

Total cellular RNA was extracted using the TRIzol method and quantified using a 4200 Bioanalyzer (Agilent Technology, Santa Clara, CA). The RIN values ranged from 8.3 to 8.7, indicating high quality of the extracted RNA. The Illumina PE150 platform was used to sequence the RNA. The obtained reads were further aligned to the reference genome of rats. The UMAP method was used for dimension reduction. Differentially expressed genes were determined using the DESeq2 package (*P* <0.05 and log_2_ (fold change) < −1 or > 1). GSEA was performed to explore the enriched signaling pathways based on the Kyoto Encyclopedia of Genes and Genomes defined gene sets. All the analyses were made by R 4.0.2 software (R Foundation for Statistical Computing, Vienna, Austria).

### Animal Modeling for DMED, AED, and BCNC

To construct the DMED model, type 1 diabetes in rats were first induced. As reported in previous studies, the rats received intraperitoneal injections of 1% streptozocin (60 mg kg^−1^; Sigma–Aldrich, St Louis, MO, USA).^[^ ^37^ ^]^ The control group received the same intraperitoneal injection of 0.1 mol L^−1^ citrate phosphate buffer. On the third day after the streptozocin injection, the fasting glucose level was determined. Rats with fasting glucose > 16.7 mmol L^−1^ were considered diabetic and were then challenged with apomorphine hypodermically (100 µg Kg^−1^; Sigma–Aldrich). Diabetic rats without erections were assigned to the DMED group. To establish the AED model, male rats aged 18 – 20 months were used. If these rats displayed normal erection under apomorphine challenge, they were assigned to the ANED group; otherwise, they were assigned to the AED group. The surgical procedure of BCNC was performed as the previous study.^[^ ^36^ ^]^ The bilateral cavernous nerves were crushed using an ultra‐fine straight hemostat with full tip closure for 30 s. The control group received the same surgical procedures without nerve crushing.

### Erectile Function Evaluation

To assess erectile function, the ICP and AUC were measured in mice and rats using a BL‐420F functional biological experiment system (Chengdu TME Technology Co., Ltd., Chengdu, China). In addition, the MAP was further evaluated in rats. Briefly, the animals were first anesthetized by isoflurane and then the skin overlying the penis was removed. Next, the left penile crus was gently exposed. A 24‐G needle was inserted into the left penile crus, which was linked with a pressure transducer to record the ICP and AUC. For rats, the left carotid artery was exposed and cannulated with a pressure transducer to record the MAP. The detailed process of assessing erectile function can be found in previous studies^[^ ^36^ ^]^ and in the supplemental files (Figure S15, Supporting Information).

### Hematoxylin‐Eosin Staining, Masson's Trichrome Staining, IHC, and Immunofluorescence Staining

Following routine dehydration and paraffin‐embedding procedures, tissue samples, which were cut into 3–5 µm sections from the mid‐shaft of the penis, were mounted on slides and dried. Next, the tissue slides were prepared for hematoxylin‐eosin (H&E) staining, Masson's trichrome staining, and IHC. Fibrosis was determined by staining tissue samples with Masson's trichrome (BA4079A, Baso Diagnostics Inc., Zhuhai, Guangdong Province, China) according to the manufacturer's protocol. For IHC and immunofluorescence, the sections were incubated with primary antibodies at 4 °C overnight. The detailed information of used antibodies was showed in Table S2 (Supporting Information). The sections of IHC and immunofluorescence were imaged with a digital camera (Vectra Polaris, PerkinElmer, Waltham, MA, USA)and Qupath software (0.4.0) were used for image analysis.

### WB Analysis

The collected cells and penile tissues were treated with radio immunoprecipitation assay (RIPA) lysis buffer (MA0151, Meilunbio, Dalian, Liaoning Province, China) to extract the protein. The proteins were wet‐transferred to polyvinylidene fluoride membranes (Millipore Corporation, Billerica, MA, USA). After blocking in 5% bovine serum albumin for 1 h at room temperature, the membranes were incubated with antibodies at 4 °C overnight. The detailed information on the antibodies used is showed in Table S3 (Supporting Information). The detection of protein signals was performed using an enhanced chemiluminescence detection system (Thermo Fisher Scientific, Waltham, MA, USA).

### Real‐Time Quantitative Polymerase Chain Reaction (RT‐qPCR)

Total RNA was extracted from the corpus cavernosum and cultured cells using TRNzol Universal (DP424, TIANGEN, Beijing, China) and then reversely transcribed to cDNA with a FastQuant RT Kit with gDNase (KR106, TIANGEN). The extraction and amplification of mRNA were performed according to the manufacturer's protocol. The relative mRNA expression of the target genes was normalized using the 2^−∆∆CT^ method. The sequences of primers used in RT‐qPCR are provided in Table S4 (Supporting Information).

### Primary CCSMC Isolation

Primary CCSMC was isolated from the rat penis according to one previous study.^[^ ^38^ ^]^ In brief, fresh corpus cavernosum was cut into 1–2 mm^3^ small pieces, and subsequently treated with 0.5% collagenase I (SCR103, Sigma‐Aldrich) for 3 h at 37 °C. 10% fetal bovine serum (FBS) in DMEM was used to terminate the digestion. A cell strainer (200 Screen Mesh) was used to filter the solution, followed by centrifugation at 12 000 g for 10 min at 4 °C. The obtained cells were washed twice with 15 mL of buffer and then cultured in DMEM at 37 °C and 5% CO_2_. After 24 h, the spindle smooth muscle cells were observed to adhere to the cell culture dish, which was cultured and used for further experiments. To characterize the extracted primary cells, cell morphology, IHC and immunofluorescence of α‐SMA, calponin, and vimentin were observed (Figure S16, Supporting Information). The high expression of α‐SMA and calponin supported that the extracted cells were mainly smooth muscle cells.

### Statistical Analyses

The pathological pictures were quantitatively analyzed by Image‐Pro Plus version 6.0 (Media Cybernetics Inc., Rockville, MD, USA). The relative expression of the proteins of interest was calculated as the grayscale ratio of the protein to tubulin, and the results were analyzed with GraphPad Prism 8.0 software (GraphPad Software, San Diego, CA, USA) and presented as mean ± standard deviation (SD). A difference was considered to be statistically significant when *P* <0.05.

### Ethnic Statement

The human samples were collected under the guidance of Ethics Committee of Shanghai General Hospital (License No. 2021SQ259) and the criteria set by the Declaration of Helsinki. Informed consent was obtained from all subjects in the original study. All experiments were performed in accordance with relevant guidelines and regulations. The mice and rats were housed and cared for under strict guidelines, and this study was approved by the Animal Care and Use Committee at the University of Texas Health Science Center and Animal Ethics Committee of West China Hospital, Sichuan University.

## Conflict of Interest

The authors declare no conflict of interest.

## Author Contributions

Y.X. and F.Q. contributed equally to this work. F.Q., S.Z.W., X.L.Y., J.L., and J.H.Y. performed the experiments. Y.X. contributed to the statistical analysis, figure preparation and writing the manuscript. Y.X., F.X.Z., and C.J.W. participated in the article screening and critical revision of the manuscript. J.H.Y. conceived this study, participated in its design and coordination, and helped draft the manuscript. All authors read and approved the final manuscript.

## Supporting information

Supporting Information

## Data Availability

The data that support the findings of this study are available on request from the corresponding author. The data are not publicly available due to privacy or ethical restrictions.
