# Supplementary material for: Targeting Adenosine A2b Receptor Promotes Penile Rehabilitation of Refractory Erectile Dysfunction
Source: Adv Sci (Weinh). 2024 Jun 14;11(30):2306514. doi: 10.1002/advs.202306514 (PMC11321695; doi:10.1002/advs.202306514)
Supplement: Supplementary file 1 — Supporting Information [file ADVS-11-2306514-s001.docx]

**Supplemental materials**

1. **Table S1：Significant enriched pathways by GSEA** (**Page 4-6**).

NES: normalized enrichment score; GSEA: Gene Set Enrichment Analysis.

1. **Table S2：Antibodies in immunohistochemistry staining** (**Page 7**).
2. **Table S3：Antibodies in western blot experiments** (**Page 8**).
3. **Table S4：Primer sequences** (**Page 9**).
4. **Figure S1：Adenosine levels detected by HPLC** (**Page 10**).

Red: ADA^-/-^ mice; Blue: wide type mice.

1. **Figure S2：Expression of F4/80 in corpus cavernosum** (**Page 11**).

F4/80 is a marker of macrophage. Scale bars = 500 μm.

1. **Figure S3：Colocalization of adenosine A2b receptor and F4/80 in corpus cavernosum** (**Page 12**).

F4/80 is a marker of macrophage. Scale bars = 50 μm or 20 μm.

1. **Figure S4：Colocalization of adenosine A2b receptor and CD31 in corpus cavernosum** (**Page 13**).

CD31 is a marker of endothelial cell. Scale bars = 50 μm or 20 μm.

1. **Figure S5：Influence of NECA (30 μM) stimulation on the TGF-β/Smad pathway** (**Page 14**).

Representative WB protein bands of TGF-β, Smad2/3, p-Smad2/3, α-SMA, and Tubulin were displayed on the left panel. The results of statistical analysis were showed on the right panel. N = 3. Statistical analysis was performed using Student-t test.

1. **Figure S6：NECA stimulations up-regulate HIF-1α, and down-regulates PDE5** (**Page 15**).

The A7r5 cells were stimulated using different NECA concentrations (0 μM, 0.1 μM, 0.3 μM, 1 μM, 3 μM, and 10 μM). Relative mRNA expressions of HIF-1α, PDE5, eNOS, and TGF-β were determined using PCR. N = 3. Statistical analysis was performed using ANOVA. **P* < 0.05, ***P* < 0.01, ****P* < 0.001.

1. **Figure S7：Results of dimension reduction by Uniform Manifold Approximation and Projection method** (**Page 16**).

The control group was from five samples cultured under hypoxia (1% O2 for 24 hours). The NECA stimulation group was from five samples cultured under hypoxia (1% O2 for 24 hours) and NECA (10 μM).

1. **Figure S8：Significant enriched pathways related to HIF-1 signaling pathways** (**Page 17**).

The control group was from five samples cultured under hypoxia (1% O2 for 24 hours). The NECA stimulation group was from five samples cultured under hypoxia (1% O2 for 24 hours) and NECA (10μM). To investigate potential pathways mediating the effects of NECA, Gene Set Enrichment Analysis (GSEA) was used. The predefined gene sets were from Kyoto Encyclopedia of Genes and Genomes (KEGG). Significant enriched pathways were defined as *P* < 0.05 and q value < 0.25. HIF-1α related pathways such as VEGF signaling pathway (Figure S5A), lysosome (Figure S5B), efferocytosis (Figure S5C), and metabolism (propanoate metabolism in Figure 5D, and ether lipid metabolism in Figure S5E) were enriched.

1. **Figure S9：Expressions of HIF-related molecules in single-cell sequencing** (**Page 18**).

The expressions of these molecules in human penile cavernous tissue were detected via single-cell sequencing. It was found that HIF-1α and HIF-2α were significantly up-reregulated in ED patients (Figure S6A-S6B). In addition, significant differences of LDHA, HO-1, and VEGF (all *P* < 0.0001) and a marginal significance of BNIP3 (*P* = 0.061) were also identified between normal individuals and patients with ED (Figure S6D-S6F).

1. **Figure S10：Expressions of HIF-related molecules under the stimulation of NECA** (**Page 19-20**).

The A7r5 cells were treated with normoxia (21% O2)/hypoxia (1% O2) and/or stimulated by NECA (3 μM and 10 μM) for 24 hours. The mRNAs were detected using qRT-PCR. As shown in Figure S7A, hypoxia, instead of NECA, can up-regulate the expression of HIF-2α. Thus, HIF-2α may not be the down-stream molecule of adenosine receptor pathways. In addition, we also found that NECA (3 μM and 10 μM) can up-regulate the expression of BNIP3 under hypoxia rather than normoxia (Figure S7B). Similarly, NECA stimulation (10 μM) under hypoxia can elevate the expression of LDHA and HO-1 (Figure S7C-S7D). Of note, the expression of HO-1 under normoxia displayed an upward trend, but it did not reach the significance threshold after adjusting for multiple testing (Bonferroni method). In Figure S7E, VEGF was significantly increased under hypoxia. This trend became more obvious when treated with NECA (3 μM and 10 μM). However, the stimulation of NECA alone cannot increase the expression of VEGF whether under hypoxia or normoxia. N = 3. Statistical analysis was performed using ANOVA. **P* < 0.05, ***P* < 0.01, ****P* < 0.001.

1. **Figure S11：Representative immunochemistry images of A1, A2a, A2b, and A3 receptors in three refractory ED models** (**Page 21**).

Original magnification×200 (scale bars = 50 μm).

1. **Figure S12：The adenosine levels in penis after Bay 60-6583 treatment in three refractory ED models** (**Page 22**).

DMED: diabetes mellitus related erectile dysfunction; AED: aging related erectile dysfunction; ANED: aged but no erectile dysfunction; BCNC: bilateral cavernous nerve crush related erectile dysfunction. N = 8. Statistical analysis was performed using ANOVA.

1. **Figure S13：The cAMP levels after Bay 60-6583 treatment in three refractory ED models** (**Page 23**).

DMED: diabetes mellitus related erectile dysfunction; AED: aging related erectile dysfunction; ANED: aged but no erectile dysfunction; BCNC: bilateral cavernous nerve crush related erectile dysfunction. N = 8. Statistical analysis was performed using Student-t test. **P* < 0.05.

1. **Figure S14：The AUCs of BCNC rats after DIP treatment** (**Page 24**).

AUC: area under the curve; BCNC: bilateral cavernous nerve crush related erectile dysfunction; DIP: dipyridamole. N = 6. Statistical analysis was performed using ANOVA. **P* < 0.05.

1. **Figure S15：Detailed process of evaluating the erectile function** (**Page 25**).
2. **Figure S16：Immunohistochemistry and immunofluorescence of α-SMA, calponin, and vimentin in CCSMC** (**Page 26**).

The expression of α-SMA (A), calponin (B), and vimentin (C) were determined. Immunofluorescence of DAPI (blue) and α-SMA, calponin, and vimentin (green) were stained in CCSMC. Original magnification×400 (scale bars = 20 μm).

**Table S1: Significant enriched pathways by GSEAs**

| **ID** | **Description** | **Set size** | **NES** | **P value** | **FDR** | **q value** |
| --- | --- | --- | --- | --- | --- | --- |
| rno00563 | Glycosylphosphatidylinositol (GPI)-anchor biosynthesis | 24 | -1.538 | 0.0193 | 0.1154 | 0.0801 |
| rno05415 | Diabetic cardiomyopathy | 193 | 1.361 | 0.0440 | 0.1397 | 0.0969 |
| rno05020 | Prion disease | 241 | 1.341 | 0.0468 | 0.1438 | 0.0997 |
| rno04961 | Endocrine and other factor-regulated calcium reabsorption | 47 | 1.448 | 0.0479 | 0.1438 | 0.0997 |
| rno04668 | TNF signaling pathway | 99 | 1.400 | 0.0382 | 0.1278 | 0.0887 |
| rno04080 | Neuroactive ligand-receptor interaction | 185 | 1.533 | 0.0150 | 0.1154 | 0.0801 |
| rno05012 | Parkinson disease | 243 | 1.333 | 0.0489 | 0.1438 | 0.0997 |
| rno05417 | Lipid and atherosclerosis | 171 | 1.435 | 0.0292 | 0.1217 | 0.0844 |
| rno04022 | cGMP-PKG signaling pathway | 138 | 1.681 | 0.0060 | 0.1128 | 0.0782 |
| rno04610 | Complement and coagulation cascades | 62 | 1.512 | 0.0285 | 0.1217 | 0.0844 |
| rno05202 | Transcriptional misregulation in cancer | 151 | 1.383 | 0.0379 | 0.1278 | 0.0887 |
| rno00310 | Lysine degradation | 59 | -1.544 | 0.0114 | 0.1154 | 0.0801 |
| rno01230 | Biosynthesis of amino acids | 70 | 1.466 | 0.0360 | 0.1278 | 0.0887 |
| rno05200 | Pathways in cancer | 447 | 1.363 | 0.0251 | 0.1154 | 0.0801 |
| rno04915 | Estrogen signaling pathway | 99 | 1.615 | 0.0115 | 0.1154 | 0.0801 |
| rno04550 | Signaling pathways regulating pluripotency of stem cells | 108 | 1.487 | 0.0224 | 0.1154 | 0.0801 |
| rno04390 | Hippo signaling pathway | 134 | 1.700 | 0.0061 | 0.1128 | 0.0782 |
| rno04024 | cAMP signaling pathway | 171 | 1.407 | 0.0327 | 0.1278 | 0.0887 |
| rno03010 | Ribosome | 152 | 1.902 | 0.0012 | 0.077 | 0.0534 |
| rno05217 | Basal cell carcinoma | 45 | 1.458 | 0.0481 | 0.1438 | 0.0997 |
| rno05022 | Pathways of neurodegeneration - multiple diseases | 425 | 1.327 | 0.0368 | 0.1278 | 0.0887 |
| rno05010 | Alzheimer disease | 341 | 1.367 | 0.0380 | 0.1278 | 0.0887 |
| rno04371 | Apelin signaling pathway | 121 | 1.622 | 0.0146 | 0.1154 | 0.0801 |
| rno04925 | Aldosterone synthesis and secretion | 83 | 1.592 | 0.0160 | 0.1154 | 0.0801 |
| rno04728 | Dopaminergic synapse | 114 | 1.487 | 0.0251 | 0.1154 | 0.0801 |
| rno04640 | Hematopoietic cell lineage | 64 | 1.657 | 0.0136 | 0.1154 | 0.0801 |
| rno04974 | Protein digestion and absorption | 80 | 1.452 | 0.0347 | 0.1278 | 0.0887 |
| rno04261 | Adrenergic signaling in cardiomyocytes | 130 | 1.711 | 0.0072 | 0.1128 | 0.0782 |
| rno04916 | Melanogenesis | 73 | 1.709 | 0.0080 | 0.1128 | 0.0782 |
| rno04934 | Cushing syndrome | 127 | 1.689 | 0.0084 | 0.1128 | 0.0783 |
| rno04713 | Circadian entrainment | 81 | 1.600 | 0.0159 | 0.1154 | 0.0801 |
| rno04062 | Chemokine signaling pathway | 139 | 1.552 | 0.0170 | 0.1154 | 0.0801 |
| rno04725 | Cholinergic synapse | 91 | 1.540 | 0.0197 | 0.1154 | 0.0801 |
| rno04540 | Gap junction | 69 | 1.551 | 0.0214 | 0.1154 | 0.0801 |
| rno04926 | Relaxin signaling pathway | 110 | 1.467 | 0.0238 | 0.1154 | 0.0801 |
| rno04927 | Cortisol synthesis and secretion | 53 | 1.556 | 0.0250 | 0.1154 | 0.0801 |
| rno04935 | Growth hormone synthesis, secretion and action | 101 | 1.462 | 0.0255 | 0.1154 | 0.0801 |
| rno04971 | Gastric acid secretion | 56 | 1.521 | 0.0302 | 0.1239 | 0.0860 |
| rno04151 | PI3K-Akt signaling pathway | 294 | 1.623 | 0.0033 | 0.1128 | 0.0782 |
| rno04066 | HIF-1 signaling pathway | 103 | 1.374 | 0.0416 | 0.1362 | 0.0945 |
| rno04144 | Endocytosis | 242 | 1.415 | 0.0356 | 0.1278 | 0.0887 |
| rno05171 | Coronavirus disease - COVID-19 | 222 | 1.714 | 0.0023 | 0.1128 | 0.0782 |
| rno05031 | Amphetamine addiction | 58 | 1.442 | 0.0490 | 0.1438 | 0.0997 |
| rno04350 | TGF-beta signaling pathway | 93 | 1.538 | 0.0194 | 0.1154 | 0.0801 |
| rno04726 | Serotonergic synapse | 89 | 1.434 | 0.0327 | 0.1278 | 0.0887 |
| rno04929 | GnRH secretion | 57 | 1.445 | 0.0450 | 0.1415 | 0.0982 |
| rno04722 | Neurotrophin signaling pathway | 112 | 1.566 | 0.0151 | 0.1154 | 0.0801 |
| rno04910 | Insulin signaling pathway | 119 | 1.384 | 0.0389 | 0.1288 | 0.0893 |
| rno00982 | Drug metabolism - cytochrome P450 | 45 | -1.456 | 0.0203 | 0.1154 | 0.0801 |
| rno05034 | Alcoholism | 126 | 1.561 | 0.0145 | 0.1154 | 0.0801 |
| rno04060 | Cytokine-cytokine receptor interaction | 165 | 1.693 | 0.0059 | 0.1128 | 0.0782 |
| rno05132 | Salmonella infection | 233 | 1.410 | 0.0336 | 0.1278 | 0.0887 |
| rno05135 | Yersinia infection | 124 | 1.509 | 0.0230 | 0.1154 | 0.0801 |
| rno04611 | Platelet activation | 108 | 1.790 | 0.0037 | 0.1128 | 0.0782 |
| rno04064 | NF-kappa B signaling pathway | 84 | 1.388 | 0.0477 | 0.1438 | 0.0997 |
| rno05144 | Malaria | 35 | 1.649 | 0.0074 | 0.1128 | 0.0782 |
| rno04061 | Viral protein interaction with cytokine and cytokine receptor | 45 | 1.660 | 0.0113 | 0.1154 | 0.0801 |
| rno00565 | Ether lipid metabolism | 39 | -1.565 | 0.0162 | 0.1154 | 0.0801 |
| rno00592 | alpha-Linolenic acid metabolism | 20 | -1.596 | 0.0337 | 0.1278 | 0.0887 |
| rno05412 | Arrhythmogenic right ventricular cardiomyopathy | 75 | 1.564 | 0.0201 | 0.1154 | 0.0801 |
| rno04142 | Lysosome | 117 | -1.709 | 0.0053 | 0.1128 | 0.0782 |
| rno04380 | Osteoclast differentiation | 110 | 1.520 | 0.0213 | 0.1154 | 0.0801 |
| rno04020 | Calcium signaling pathway | 200 | 1.570 | 0.0081 | 0.1128 | 0.0782 |
| rno04520 | Adherens junction | 83 | 1.461 | 0.0279 | 0.1217 | 0.0844 |
| rno05220 | Chronic myeloid leukemia | 74 | 1.449 | 0.0359 | 0.1278 | 0.0887 |
| rno00270 | Cysteine and methionine metabolism | 44 | 1.562 | 0.0201 | 0.1154 | 0.0801 |
| rno04921 | Oxytocin signaling pathway | 131 | 1.525 | 0.0229 | 0.1154 | 0.0801 |
| rno04310 | Wnt signaling pathway | 142 | 1.513 | 0.0215 | 0.1154 | 0.0801 |
| rno05163 | Human cytomegalovirus infection | 193 | 1.451 | 0.0255 | 0.1154 | 0.0801 |
| rno00280 | Valine, leucine and isoleucine degradation | 48 | -1.532 | 0.0233 | 0.1154 | 0.0801 |
| rno05165 | Human papillomavirus infection | 293 | 1.390 | 0.0369 | 0.1278 | 0.0887 |
| rno05203 | Viral carcinogenesis | 176 | 1.353 | 0.0421 | 0.1365 | 0.0947 |
| rno05224 | Breast cancer | 121 | 1.589 | 0.0146 | 0.1154 | 0.0801 |
| rno04814 | Motor proteins | 170 | 1.658 | 0.0070 | 0.1128 | 0.0782 |
| rno05100 | Bacterial invasion of epithelial cells | 73 | 1.697 | 0.0093 | 0.1154 | 0.0801 |
| rno04010 | MAPK signaling pathway | 269 | 1.624 | 0.0033 | 0.1128 | 0.0782 |
| rno04015 | Rap1 signaling pathway | 175 | 1.642 | 0.0059 | 0.1128 | 0.0782 |
| rno04148 | Efferocytosis | 142 | 1.462 | 0.0287 | 0.1217 | 0.0844 |
| rno05414 | Dilated cardiomyopathy | 90 | 1.547 | 0.0197 | 0.1154 | 0.0801 |
| rno04360 | Axon guidance | 157 | 1.404 | 0.0333 | 0.1278 | 0.0887 |
| rno05206 | MicroRNAs in cancer | 205 | 1.538 | 0.0103 | 0.1154 | 0.0801 |
| rno04014 | Ras signaling pathway | 199 | 1.504 | 0.0185 | 0.1154 | 0.0801 |
| rno05214 | Glioma | 68 | 1.638 | 0.0147 | 0.1154 | 0.0801 |
| rno05216 | Thyroid cancer | 35 | 1.537 | 0.0251 | 0.1154 | 0.0801 |
| rno01521 | EGFR tyrosine kinase inhibitor resistance | 72 | 1.445 | 0.0365 | 0.1278 | 0.0887 |
| rno04530 | Tight junction | 151 | 1.839 | 0.0012 | 0.077 | 0.0534 |
| rno04740 | Olfactory transduction | 106 | 1.863 | 0.0013 | 0.077 | 0.0534 |
| rno04260 | Cardiac muscle contraction | 76 | 1.680 | 0.0120 | 0.1154 | 0.0801 |
| rno04810 | Regulation of actin cytoskeleton | 198 | 1.696 | 0.0012 | 0.077 | 0.0534 |
| rno04510 | Focal adhesion | 187 | 1.783 | 0.0012 | 0.077 | 0.0534 |
| rno04270 | Vascular smooth muscle contraction | 107 | 1.653 | 0.0100 | 0.1154 | 0.0801 |
| rno04630 | JAK-STAT signaling pathway | 116 | 1.466 | 0.0271 | 0.1209 | 0.0839 |
| rno04670 | Leukocyte transendothelial migration | 99 | 1.718 | 0.0076 | 0.1128 | 0.0782 |
| rno04514 | Cell adhesion molecules | 120 | 1.429 | 0.0327 | 0.1278 | 0.0887 |
| rno04512 | ECM-receptor interaction | 77 | 1.416 | 0.0427 | 0.1369 | 0.0950 |
| rno05205 | Proteoglycans in cancer | 178 | 1.673 | 0.0058 | 0.1128 | 0.0782 |
| rno05219 | Bladder cancer | 35 | 1.644 | 0.0074 | 0.1128 | 0.0782 |
| rno05226 | Gastric cancer | 121 | 1.560 | 0.0182 | 0.1154 | 0.0801 |
| rno04370 | VEGF signaling pathway | 53 | 1.509 | 0.0292 | 0.1217 | 0.0844 |
| rno05215 | Prostate cancer | 85 | 1.380 | 0.0490 | 0.1438 | 0.0997 |
| rno05410 | Hypertrophic cardiomyopathy | 90 | 1.525 | 0.0197 | 0.1154 | 0.0801 |
| rno05145 | Toxoplasmosis | 93 | 1.496 | 0.0207 | 0.1154 | 0.0801 |
| rno05146 | Amoebiasis | 82 | 1.436 | 0.0370 | 0.1278 | 0.0887 |
| rno00640 | Propanoate metabolism | 30 | -1.585 | 0.0145 | 0.1154 | 0.0801 |
| rno05340 | Primary immunodeficiency | 29 | 1.623 | 0.0137 | 0.1154 | 0.0801 |

NES: normalized enrichment score; GSEA: Gene Set Enrichment Analysis.

**Table S2: Antibodies in immunohistochemistry and immunofluorescence staining**

| **Antibody** | **Concentration** | **Lot number** | **Corporation** |
| --- | --- | --- | --- |
| eNOS | 1:100 | Ab76198 | Abcam |
| α-SMA | 1:200 | Ab32595 | Abcam |
| TGF-β | 1:100 | Ab92486 | Abcam |
| HIF-1α | 1:100 | Ab2185 | Abcam |
| Adenosine A1 receptor | 1:100 | Ab82477 | Abcam |
| Adenosine A2a receptor | 1:100 | Ab3461 | Abcam |
| Adenosine A2b receptor | 1:100 | Ab229671 | Abcam |
| Adenosine A3 receptor | 1:100 | Ab203298 | Abcam |
| Calponin | 1:100 | A3734 | Abclonal |
| Myosin | 1:100 | 49349S | CST |
| CD31 | 1:500 | Ab222783 | Abcam |
| FSP-1 | 1:100 | A19109 | Abclonal |
| F4/80 | 1:200 | 30325S | CST |

**Table S3: Antibodies in western blot experiments**

| **Antibody** | **Concentration** | **Lot number** | **Corporation** |
| --- | --- | --- | --- |
| eNOS | 1:1000 | Ab76198 | Abcam |
| α-SMA | 1:1000 | Ab32595 | Abcam |
| TGF-β | 1:1000 | Ab92486 | Abcam |
| HIF-1α | 1:1000 | Ab2185 | Abcam |
| PDE5 | 1:1000 | Ab259945 | Abcam |
| A1 | 1:1000 | Ab82477 | Abcam |
| A2a | 1:1000 | Ab3461 | Abcam |
| A2b | 1:1000 | Ab229671 | Abcam |
| A3 | 1:1000 | Ab203298 | Abcam |
| Smad2/3 | 1:1000 | Ab202445 | Abcam |
| p-smad2/3 | 1:1000 | Ab272332 | Abcam |
| Tubulin | 1:2000 | 200608 | Zen BioScience |

**Table S4: Primer sequences**

| **Genes** | **Primer sequence (5'-3')** |
| --- | --- |
| A2bR | F: GCGTCCCGCTCAGGTATAAA |
|  | R: CCAATGCCAAAGGCAAGGAC |
| eNOS | F: ACAGGCATCACCAGGAAGAAG |
|  | R: CAGAGCCATACAGGATAGTCG |
| HIF-1α | F: TCAAGTCAGCAACGTGGAAG |
|  | R: TTCACAAATCAGCACCAAGC |
| α-SMA | F: TTCCTTCGTGACTACTGCTGAG |
|  | R: CAATGAAAGATGGCTGGAAGAG |
| PDE5 | F: CGAGGAGCAGCAGTCATTGGAA |
|  | R: AGAGAACGAGTCAGGGCAGTCT |
| TGF-β | F: CTGCTGACCCCCACTGATAC |
|  | R: CTGTATTCCGTCTCCTTGGTTC |
| HIF-2α | F: ACCTGGAAGGTCTTGCACTGC |
|  | R: TCACACATGATGATGAGGCAGG |
| LDHA | F: CGTCTGCCCTATCAACTTTCG |
|  | R: CTTGGATGTGGTAGCCGTTTC |
| VEGF | F: CACGACAGAAGGGGAGCAGAAAG |
|  | R: GGCACACAGGACGGCTTGAAG |
| BNIP3 | F: TGAAATAGACAGCCACAG |
|  | R: GACTTGACCAATCCCATA |
| HO-1 | F: TAAGACCGCCTTCCTGCTCAA |
|  | R: ACGGTCGCCAACAGGAAACT |
| GAPDH | F: CCTCAAGATTGTCAGCAAT |
|  | R: CCATCCACAGTCTTCTGAGT |


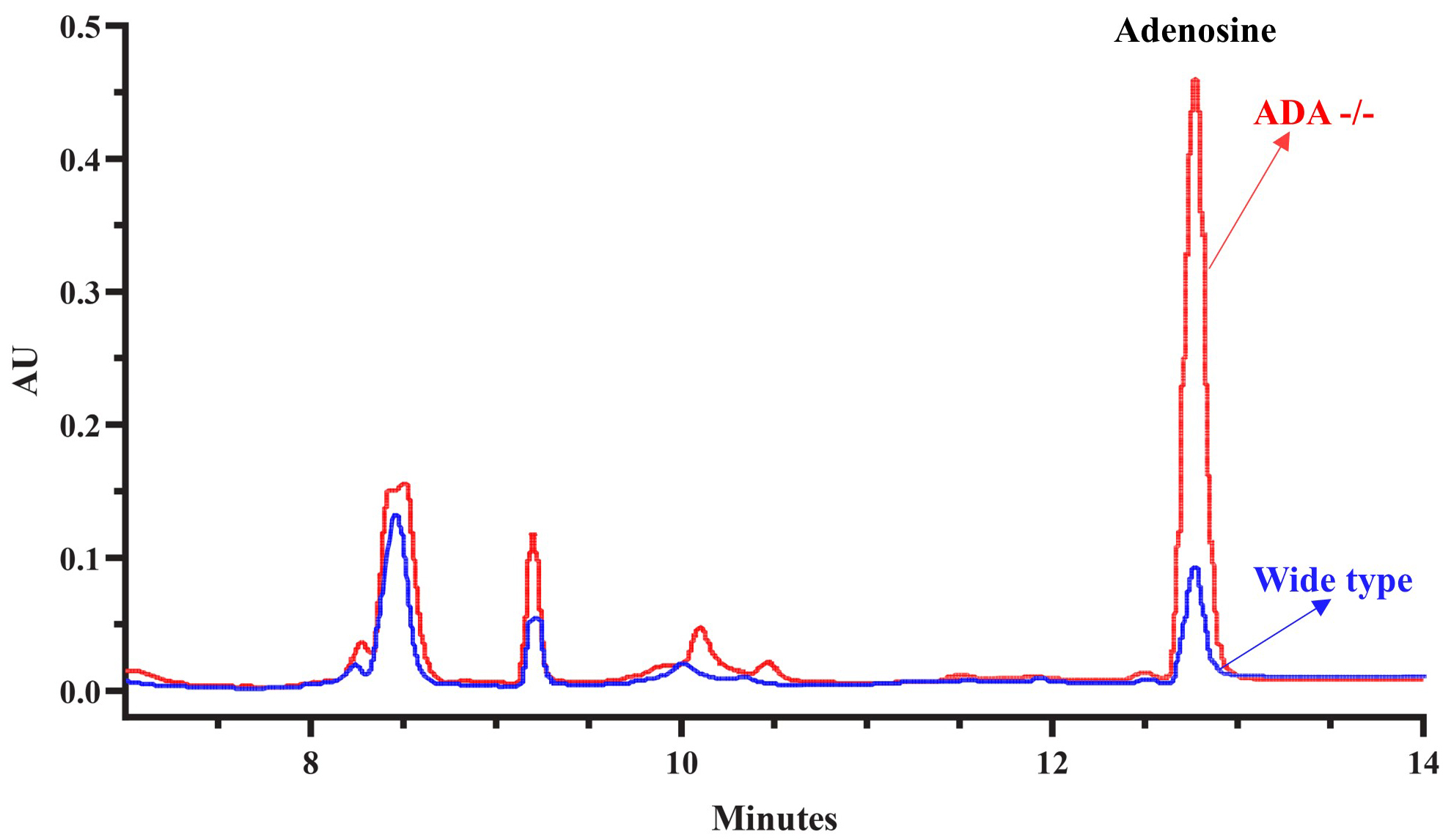


**Figure S1: Adenosine levels detected by high-performance liquid chromatography**

Red: ADA^-/-^ mice; Blue: wide type mice.


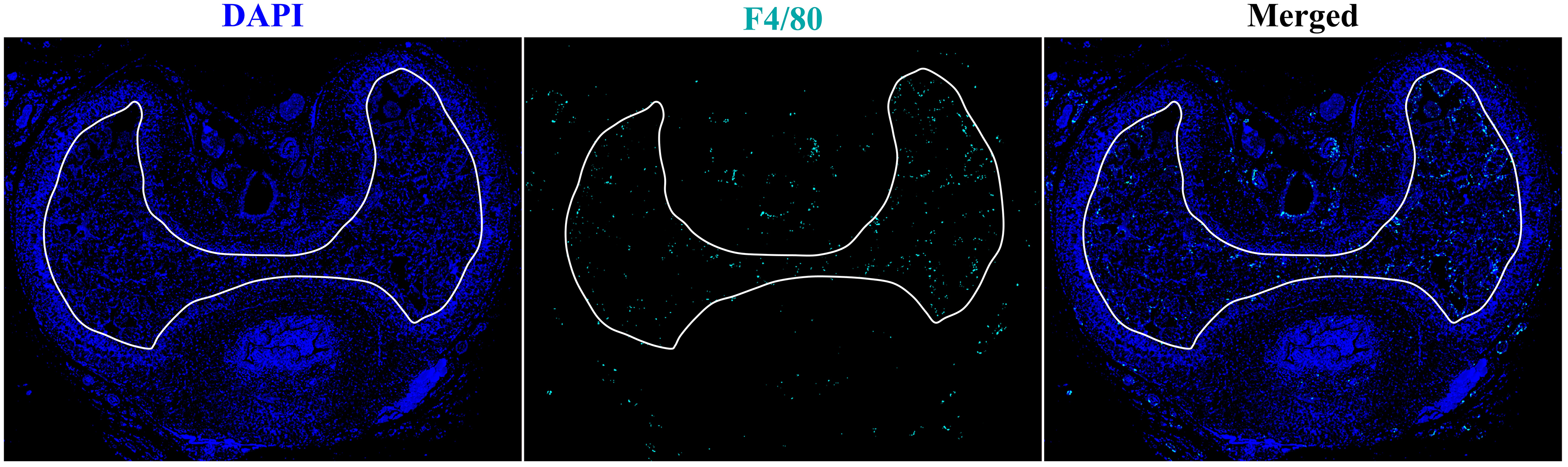


**Figure S2: Expression of F4/80 in corpus cavernosum**

F4/80 is a marker of macrophage. Scale bars = 500 μm.


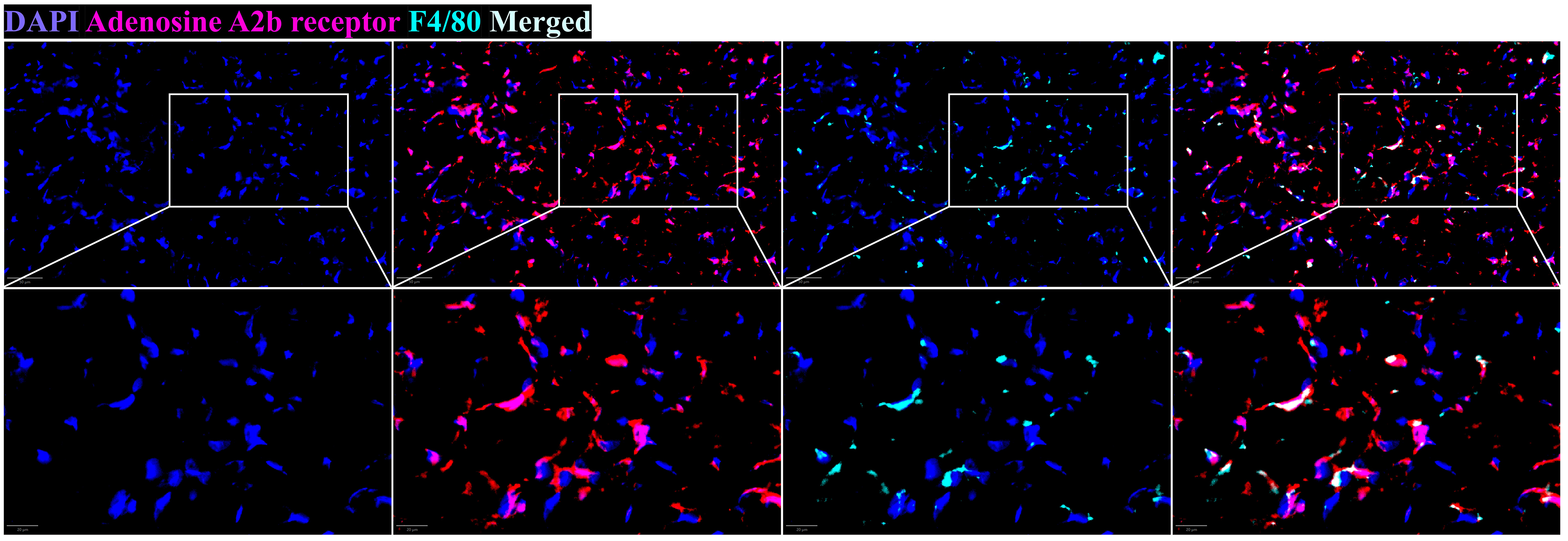


**Figure S3: Colocalization of adenosine A2b receptor and F4/80 in corpus cavernosum**

F4/80 is a marker of macrophage. Scale bars = 50 μm or 20 μm.


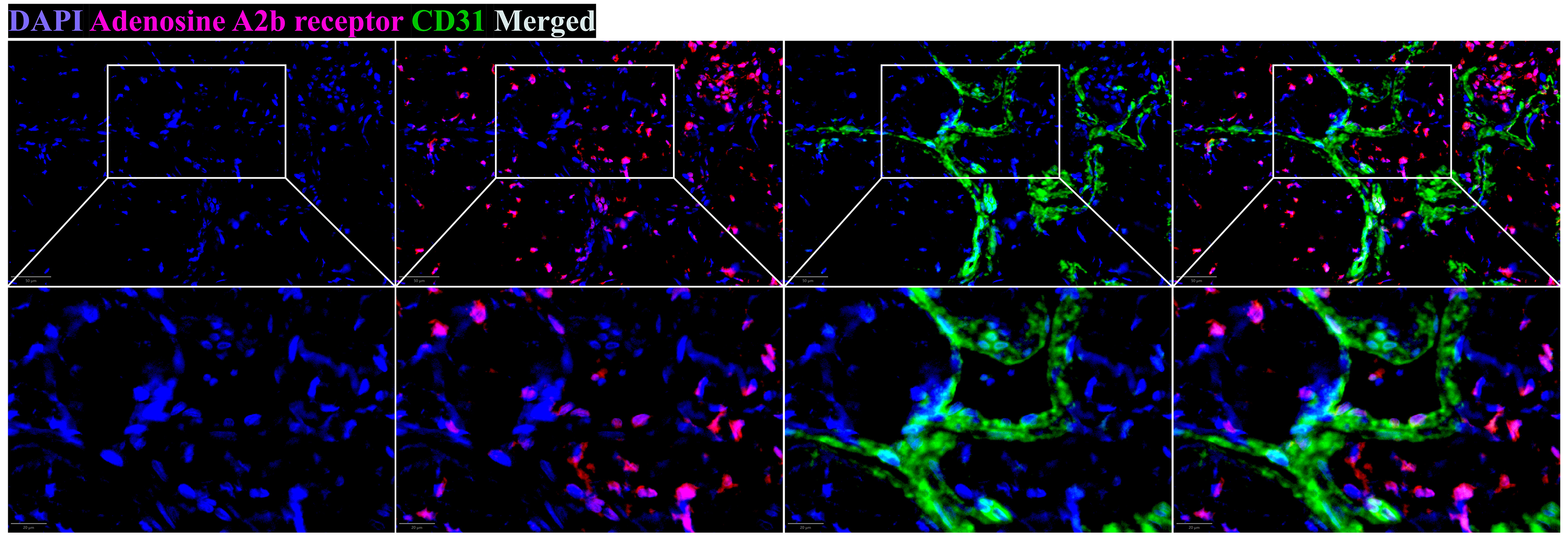


**Figure S4: Colocalization of adenosine A2b receptor and CD31 in corpus cavernosum**

CD31 is a marker of endothelial cell. Scale bars = 50 μm or 20 μm.


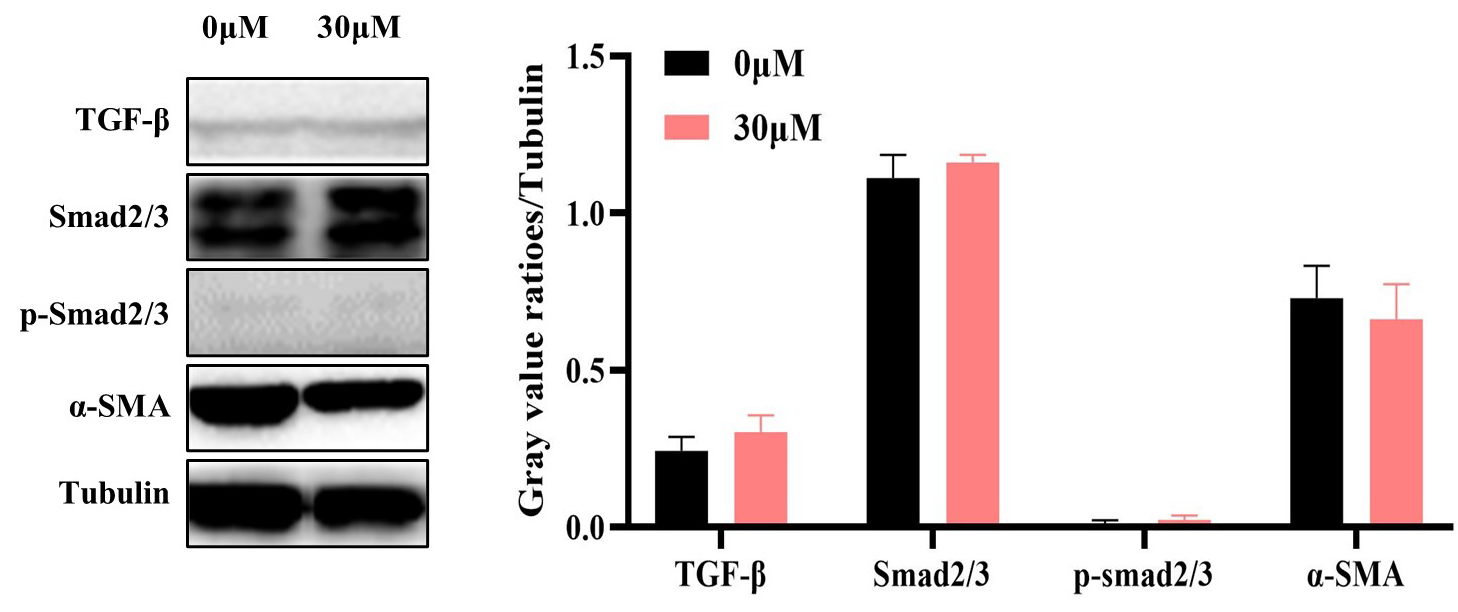


**Figure S5: Influence of NECA (30 μM) stimulation on the TGF-β/Smad pathway**

Representative WB protein bands of TGF-β, Smad2/3, p-Smad2/3, α-SMA, and Tubulin were displayed on the left panel. The results of statistical analysis were showed on the right panel. N = 3. Statistical analysis was performed using Student-t test.


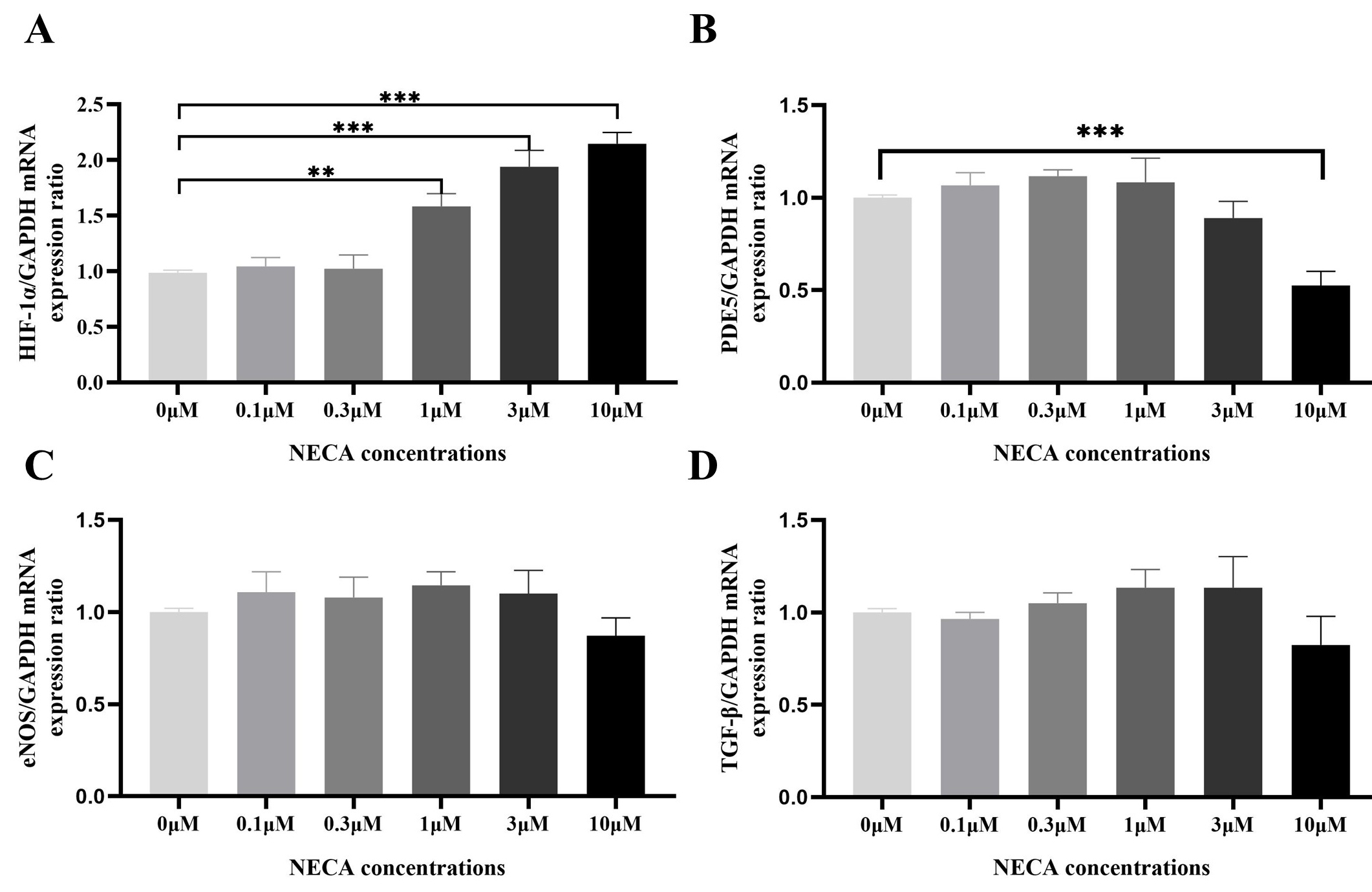


**Figure S6: NECA stimulations up-regulate HIF-1α, and down-regulates PDE5**

The A7r5 cells were stimulated using different NECA concentrations (0 μM, 0.1 μM, 0.3 μM, 1 μM, 3 μM, and 10 μM). Relative mRNA expressions of HIF-1α, PDE5, eNOS, and TGF-β were determined using PCR. N = 3. Statistical analysis was performed using ANOVA. **P* < 0.05, ***P* < 0.01, ****P* < 0.001.

**
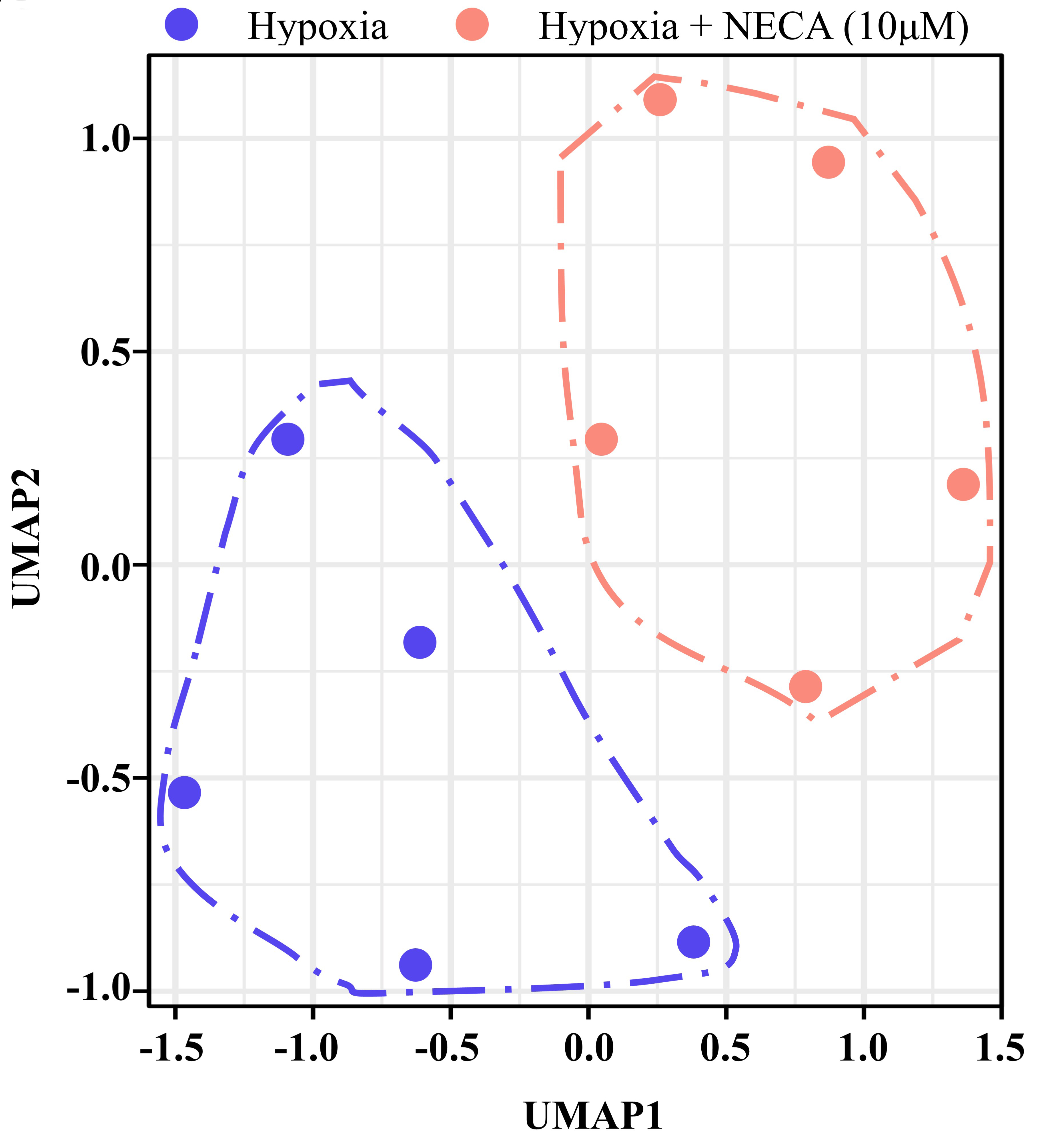
**

**Figure S7:** **Results of dimension reduction by Uniform Manifold Approximation and Projection method**

The control group was from five samples cultured under hypoxia (1% O2 for 24 hours). The NECA stimulation group was from five samples cultured under hypoxia (1% O2 for 24 hours) and NECA (10μM).


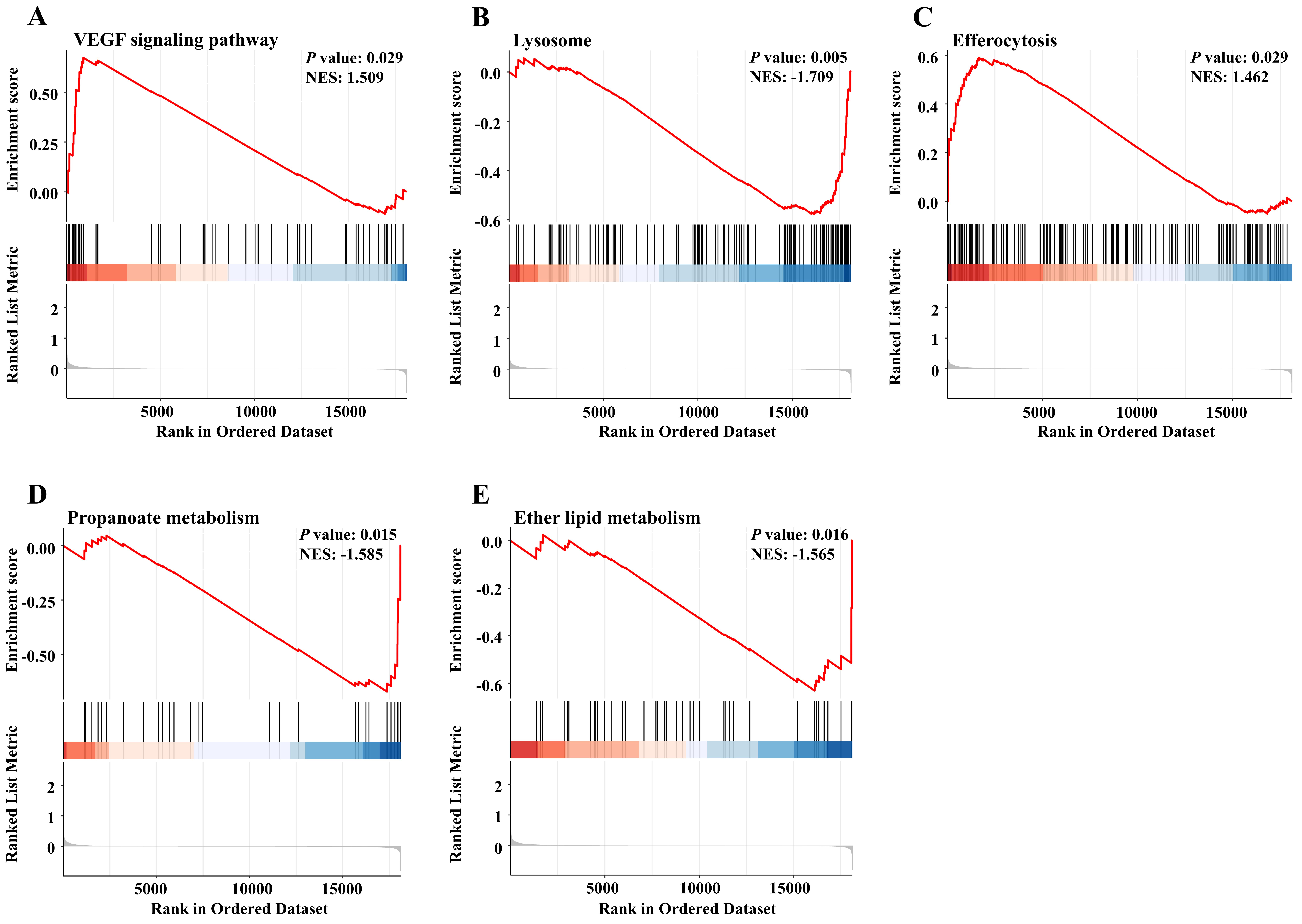


**Figure S8: Significant enriched pathways related to HIF-1 signaling pathways**

The control group was from five samples cultured under hypoxia (1% O2 for 24 hours). The NECA stimulation group was from five samples cultured under hypoxia (1% O2 for 24 hours) and NECA (10μM). To investigate potential pathways mediating the effects of NECA, Gene Set Enrichment Analysis (GSEA) was used. The predefined gene sets were from Kyoto Encyclopedia of Genes and Genomes (KEGG). Significant enriched pathways were defined as *P* < 0.05 and q value < 0.25. HIF-1α related pathways such as VEGF signaling pathway (Figure S5A), lysosome (Figure S5B), efferocytosis (Figure S5C), and metabolism (propanoate metabolism in Figure 5D, and ether lipid metabolism in Figure S5E) were enriched.





**Figure S9: Expressions of HIF-related molecules in single-cell sequencing**

The expressions of these molecules in human penile cavernous tissue were detected via single-cell sequencing. It was found that HIF-1α and HIF-2α were significantly up-reregulated in ED patients (Figure S6A-S6B). In addition, significant differences of LDHA, HO-1, and VEGF (all *P* < 0.0001) and a marginal significance of BNIP3 (*P* = 0.061) were also identified between normal individuals and patients with ED (Figure S6D-S6F).


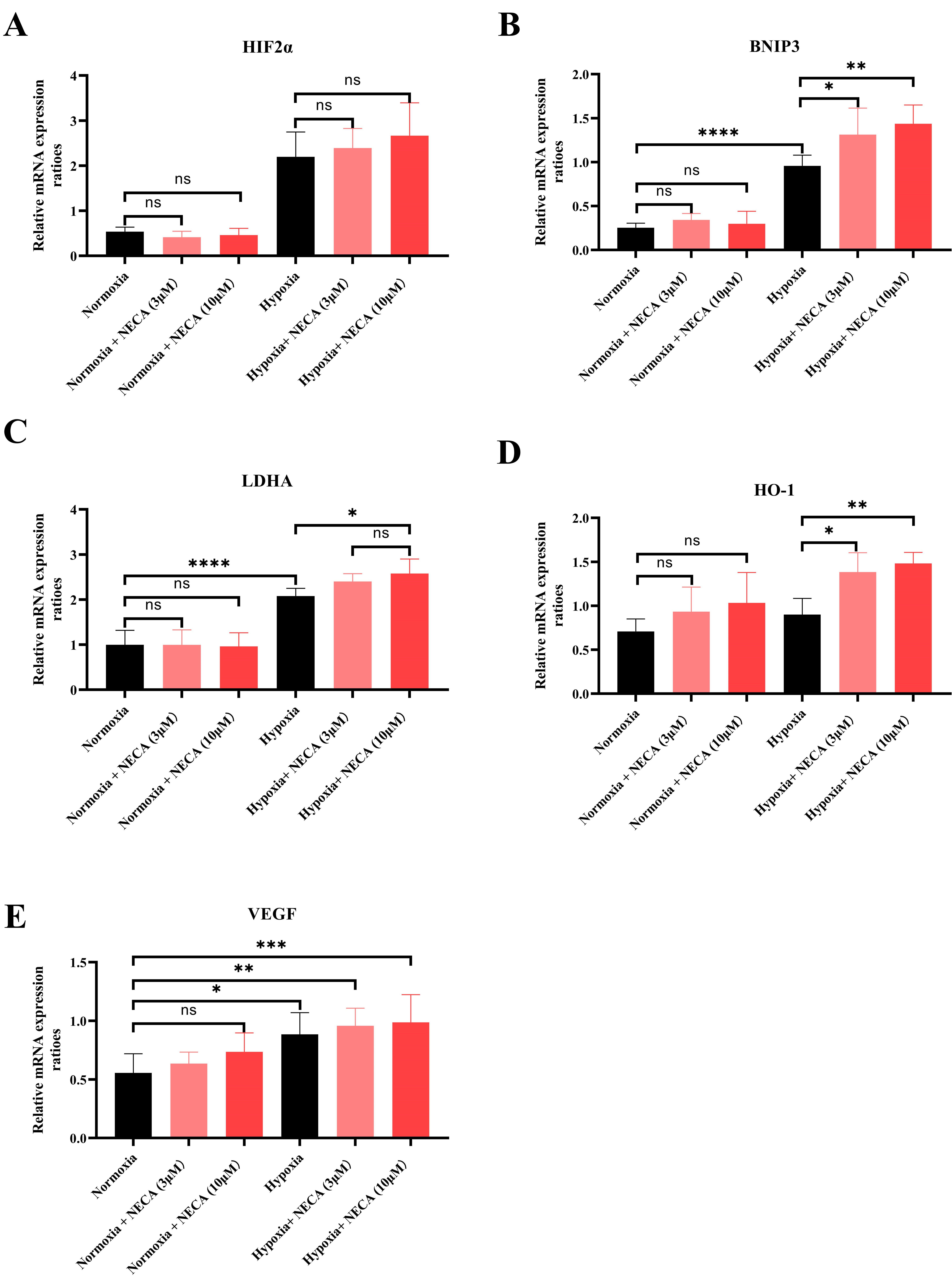


**Figure S10: Expressions of HIF-related molecules under the stimulation of NECA**

The A7r5 cells were treated with normoxia (21% O2)/hypoxia (1% O2) and/or stimulated by NECA (3 μM and 10 μM) for 24 hours. The mRNAs were detected using qRT-PCR. As shown in Figure S7A, hypoxia, instead of NECA, can up-regulate the expression of HIF-2α. Thus, HIF-2α may not be the down-stream molecule of adenosine receptor pathways. In addition, we also found that NECA (3 μM and 10 μM) can up-regulate the expression of BNIP3 under hypoxia rather than normoxia (Figure S7B). Similarly, NECA stimulation (10 μM) under hypoxia can elevate the expression of LDHA and HO-1 (Figure S7C- S7D). Of note, the expression of HO-1 under normoxia displayed an upward trend, but it did not reach the significance threshold after adjusting for multiple testing (Bonferroni method). In Figure S7E, VEGF was significantly increased under hypoxia. This trend became more obvious when treated with NECA (3 μM and 10 μM). However, the stimulation of NECA alone cannot increase the expression of VEGF whether under hypoxia or normoxia.


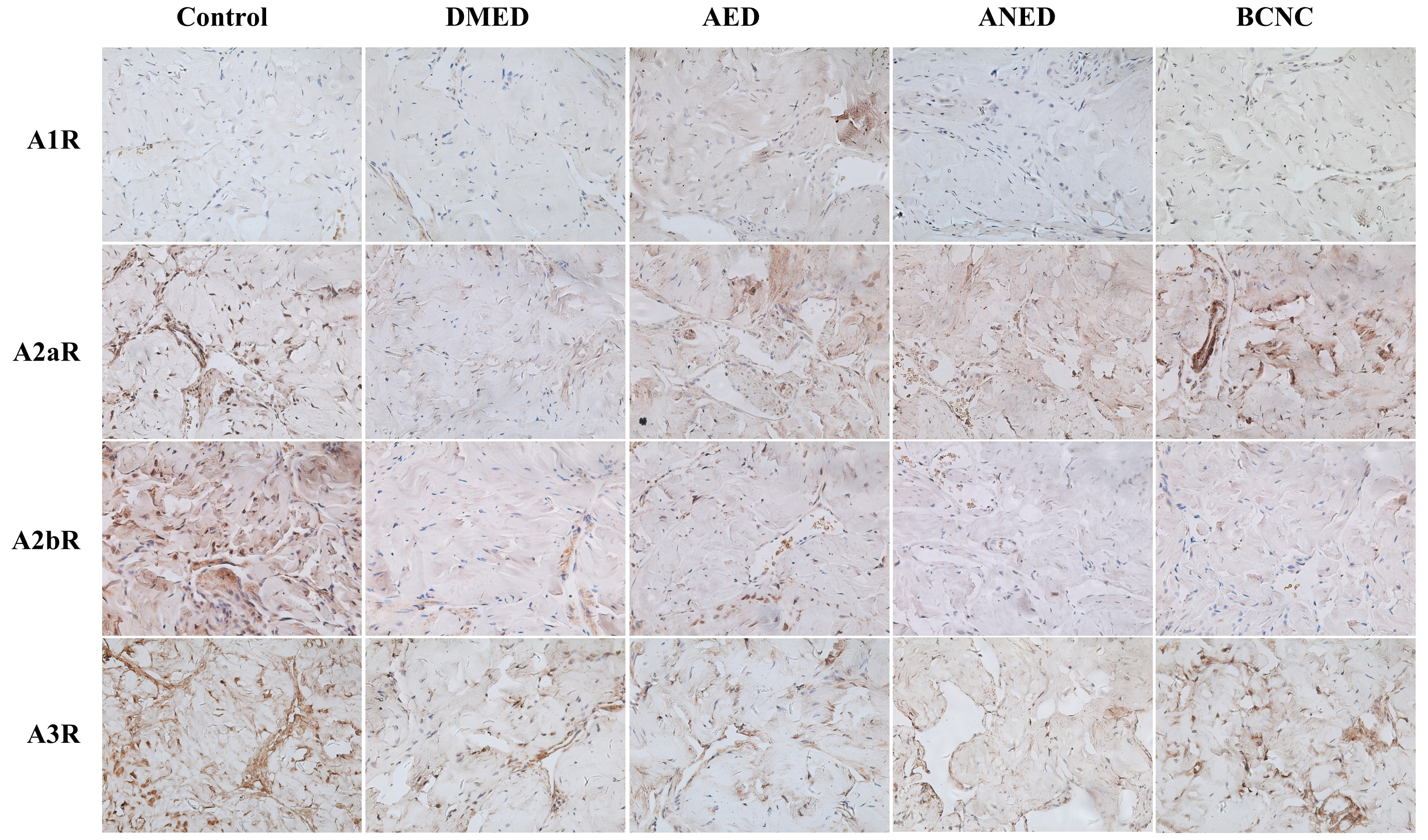


**Figure S11: Representative immunochemistry images of A1, A2a, A2b, and A3 receptors in three refractory ED models**

DMED: diabetes mellitus related erectile dysfunction; AED: aging related erectile dysfunction; ANED: aged but no erectile dysfunction; BCNC: bilateral cavernous nerve crush related erectile dysfunction. Original magnification×200 (scale bars = 50 μm).


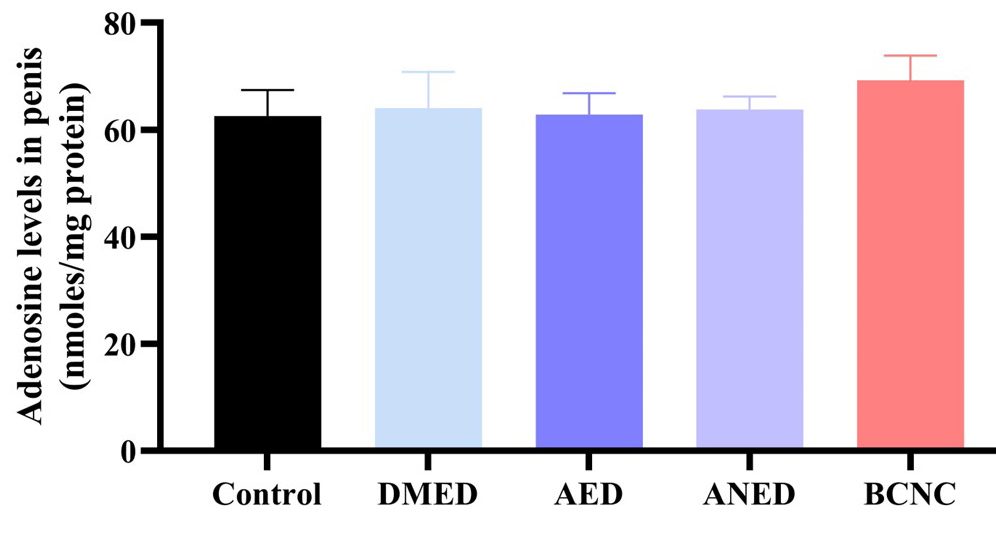


**Figure S12: The adenosine levels in penis after Bay 60-6583 treatment in three refractory ED models**

DMED: diabetes mellitus related erectile dysfunction; AED: aging related erectile dysfunction; ANED: aged but no erectile dysfunction; BCNC: bilateral cavernous nerve crush related erectile dysfunction. N = 8. Statistical analysis was performed using ANOVA.


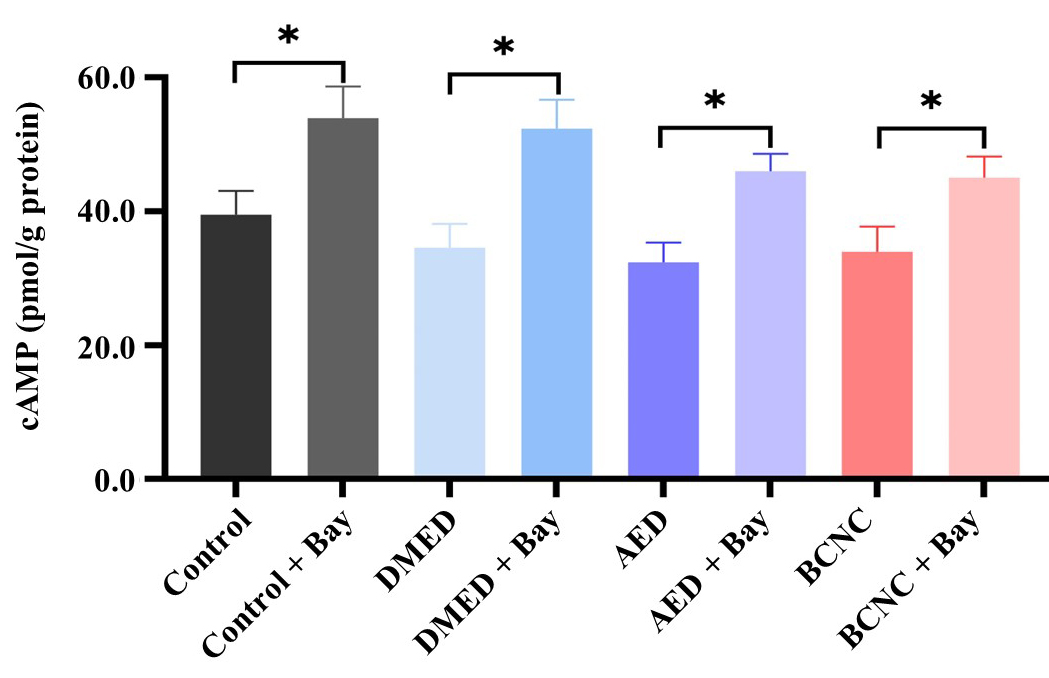


**Figure S13: The cAMP levels after Bay 60-6583 treatment in three refractory ED models**

DMED: diabetes mellitus related erectile dysfunction; AED: aging related erectile dysfunction; ANED: aged but no erectile dysfunction; BCNC: bilateral cavernous nerve crush related erectile dysfunction. N = 8. Statistical analysis was performed using Student-t test. **P* < 0.05.


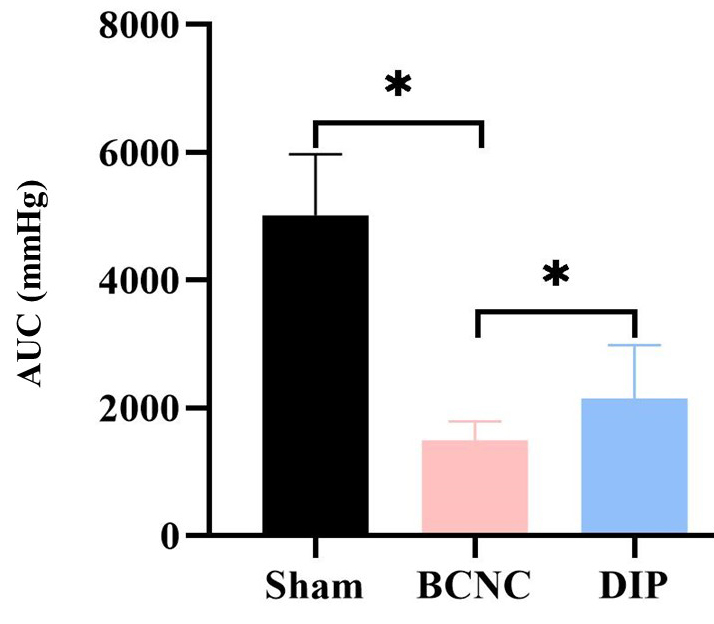


**Figure S14: The AUCs of BCNC rats after DIP treatment**

AUC: area under the curve; BCNC: bilateral cavernous nerve crush related erectile dysfunction; DIP: dipyridamole. N = 6. Statistical analysis was performed using ANOVA. **P* < 0.05.

**Detailed process of evaluating the erectile function**

The method of ICP detection in mice was performed as follows (**Figure S12**):

1) as shown in **Figure S12A**, the mice were anesthetized by inhaling 3 Vol% isoflurane (Veteasy 100% V/V; RWD life science, Shenzhen, Guangdong Province, China) in a chamber for 3–5 min and maintained anesthesia at 1–1.5 Vol% isoflurane with 0.8 L/min air as the carrier via a nose cone. Air pump (R510–25; RWD life science) was used as an air source throughout the anesthesia. After the mice was anesthetized, a low midline incision was made and the skin overlying the penis was removed.

2) as shown in **Figure 12B**, we carefully exposed the left penile crus. In this step, the penis should be carefully exposed and should not be damaged. Otherwise, it can lead to pressure leakage, biasing the results.

3) as shown in **Figure 12C**, a 24-G needle (SGJS Medical Equipment Group Co. Ltd., Luohe, Henan Province, China) filled with heparin (250 U/ml) was inserted into the left side of the penile crus and attached to a pressure transducer with polyethylene tubing filled with heparin solution for ICP recording. As you can see, a successful insertion can induce erection without leakage when injecting with adenosine. A BL-420F biological function experiment system (Chengdu TME Technology Co. Ltd.) was used for recording ICP values for this experiment.


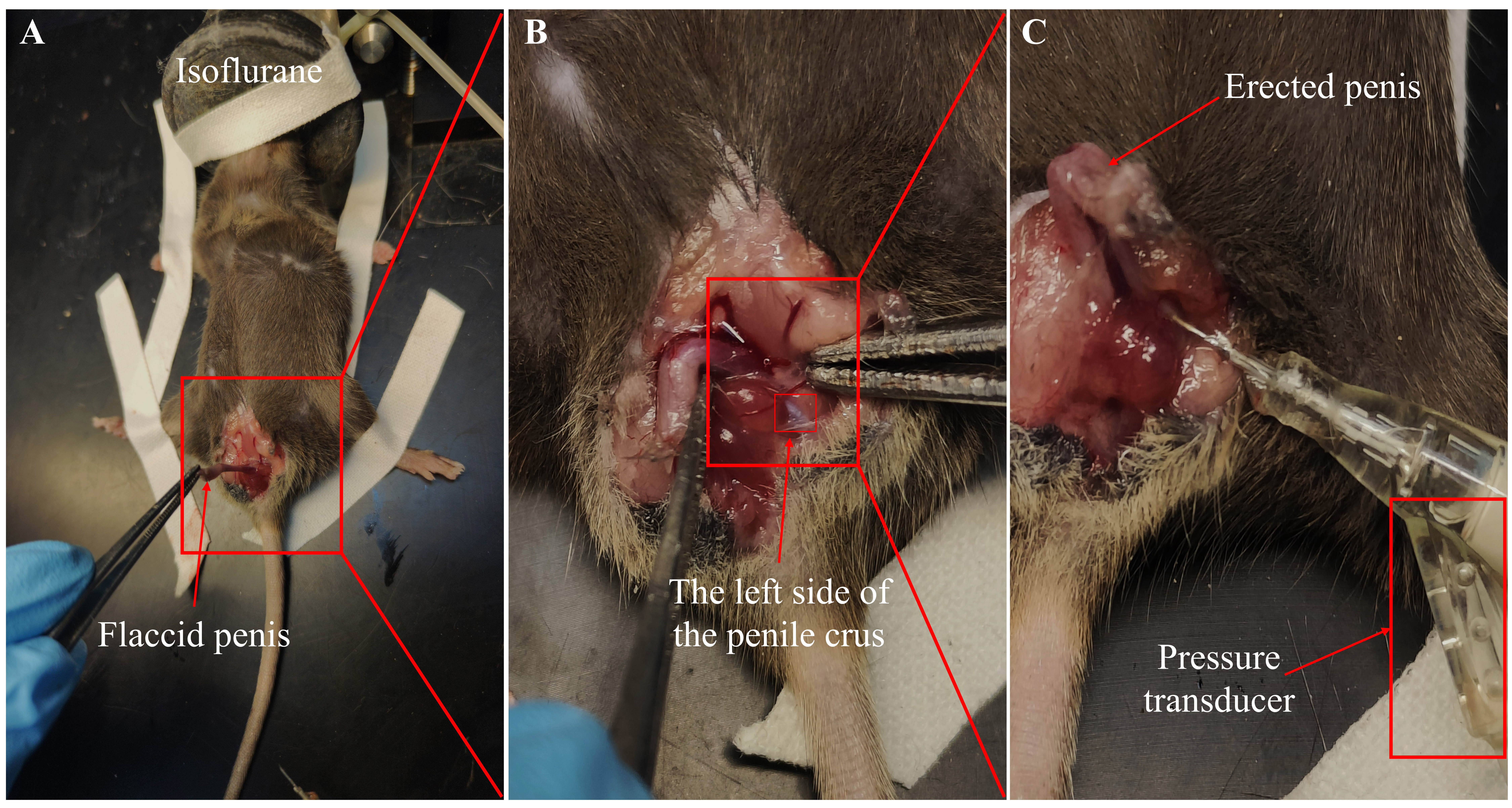


**Figure S15: The method of determining ICP in mice.**


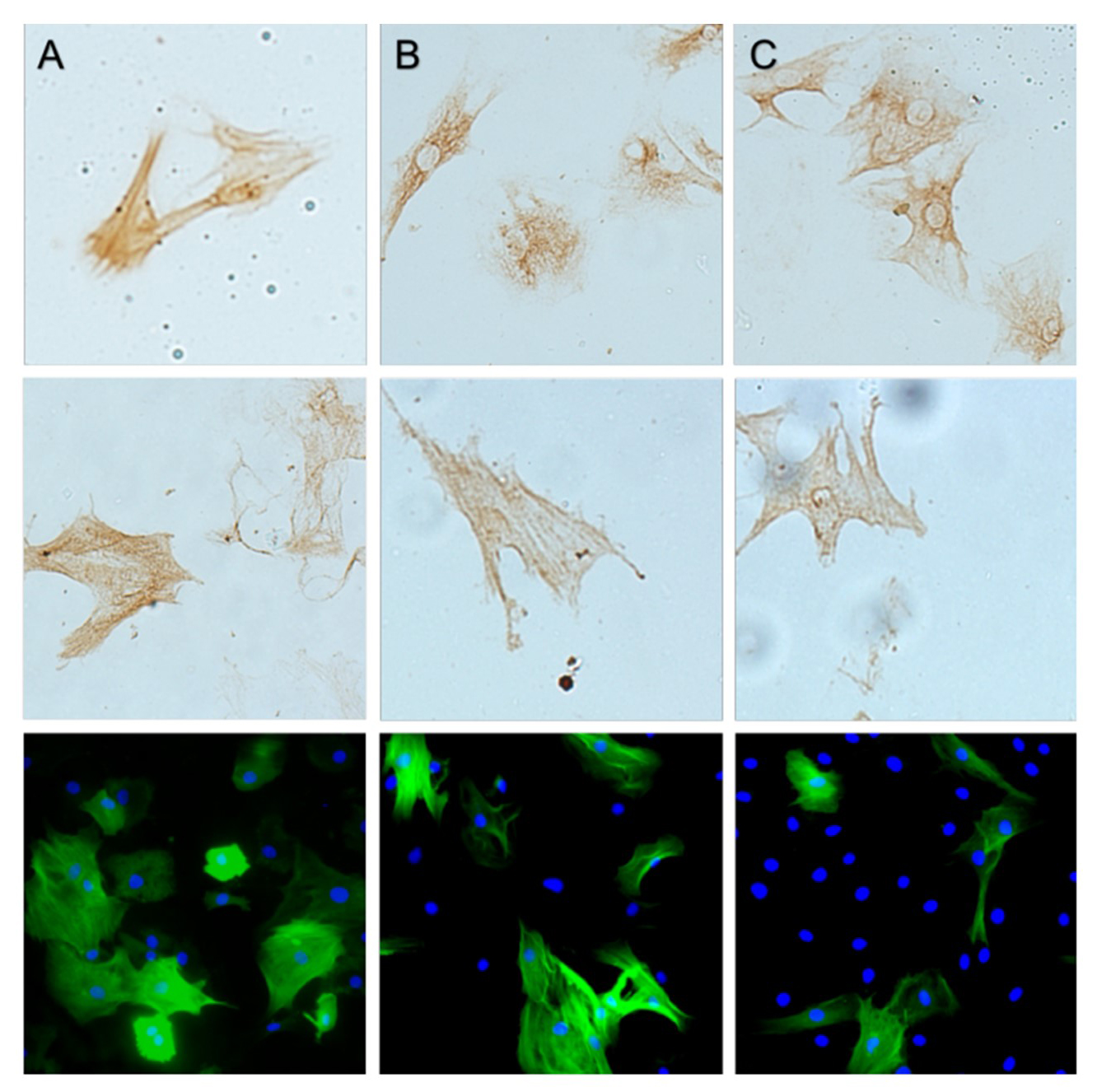


**Figure S16: Immunohistochemistry and immunofluorescence of α-SMA, calponin, and vimentin in CCSMC**

The expression of α-SMA (A), calponin (B), and vimentin (C) were determined. Immunofluorescence of DAPI (blue) and α-SMA, calponin, and vimentin (green) were stained in CCSMC. Original magnification×400 (scale bars = 20 μm).
